# Genomic signatures of vegetable and oilseed allopolyploid *Brassica juncea* and genetic loci controlling the accumulation of glucosinolates

**DOI:** 10.1111/pbi.13687

**Published:** 2021-10-01

**Authors:** Jinghua Yang, Jing Wang, Zhangping Li, Xuming Li, Zhesi He, Lili Zhang, Tongyun Sha, Xiaolong Lyu, Sheng Chen, Yuanguo Gu, Zaiyun Li, Zhongyuan Hu, Hongju He, Ian Bancroft, Mingfang Zhang

**Affiliations:** ^1^ Laboratory of Germplasm Innovation and Molecular Breeding Institute of Vegetable Science Zhejiang University Hangzhou China; ^2^ Yazhou Bay Science and Technology City Hainan Institute of Zhejiang University Yazhou District, Sanya China; ^3^ Key Laboratory of Horticultural Plant Growth and Development Ministry of Agriculture and Rural Affairs Hangzhou China; ^4^ Biomarker Technologies Corporation Beijing China; ^5^ Department of Biology University of York York UK; ^6^ School of Agriculture and Environment and the UWA Institute of Agriculture The University of Western Australia Perth WA Australia; ^7^ Xinjiang Academy of Agricultural Sciences Urumqi China; ^8^ College of Plant Science & Technology Huazhong Agricultural University Wuhan China; ^9^ Beijing Academy of Agricultural and Forestry Sciences Beijing China

**Keywords:** *Brassica juncea*, allopolyploid, structural variations, copy number variation, glucosinolates

## Abstract

Allopolyploid *Brassica juncea* crops in *Brassicaceae* are becoming increasingly revitalized as vegetables and oilseeds owing to wide adaptability and significant economic values. However, the genomic differentiation of diversified vegetables and oilseed *B. juncea* and the genetic basis underlying glucosinolates accumulation have yet to be elucidated. To address this knowledge gap, we report the sequencing of pairwise genomes of vegetable and oilseed *B. juncea* at chromosome scale. Comparative genomics analysis unveils panoramic structural variation footprints, particularly the genetic loci of *HSP20* and *TGA1* associated with abiotic and biotic stresses responses between oilseed and vegetable subgroups. We anchored two major loci of *MYB28* (*HAG1*) orthologues caused by copy number variations on A02 and A09 chromosomes using scored genomic SNPs‐based GWAS that are responsible for seed oil quality‐determining glucosinolates biosynthesis. These findings will provide valuable repertories of polyploidy genomic information enabling polyploidy genome evolution studies and precise genomic selections for crucial traits like functional components of glucosinolates in *B. juncea* crops and beyond.

## Introduction

The *Brassica* genus features distinctive vegetable and oilseed crops that are cultivated worldwide and essential for human nutrition. Data show that 71 million tonnes of vegetables and 4.7 million tonnes of oilseeds produced from plants of this genus in 2017 (http://www.fao.org/faostat/). The allopolyploid *B. juncea* (AABB) crop was originally derived from distant hybridization between *B. rapa* (AA) and *B. nigra* (BB), followed by spontaneous chromosome doubling (Nagaharu, 1935). Diversifying selection for subsequent agricultural application then formed vegetables and oilseeds from *B. juncea* (Yang *et al*., 2016, 2018), thus evolving into two highly diversified subgroups. In order to improve the quality breeding of *B. juncea* products, it is important for vegetable varieties to be enriched in beneficial glucosinolates (GSLs) compositions for health‐promoting nutrition (Verkerk *et al*., 2009), while for oilseed varieties, it is preferable to produce seeds containing high levels of polyunsaturated fatty acids and low erucic acid (Chalhoub *et al*., 2014), low levels of GSLs.

GSLs content is a major determinant of seed quality in oilseed *Brassica* crops; GSLs are known to exert anti‐nutritional effects in the residual meal used for animal feed products (Schmidt and Bancroft, 2011; Snowdon *et al*., 2007). Low GSL breeding of *B. juncea* was firstly reported to be generated from interspecific hybridization between an Indian oilseed *B. juncea* variety with a low GSL *B. rapa* line backcrossed with the Indian oilseed *B. juncea* variety (Love *et al*., 1990). This line has been subsequently incorporated into a diverse breeding pipeline for development of *B. juncea* containing low GSLs. Studies using quantitative trait loci (QTL) mapping and genome‐wide association studies (GWAS) have already been applied to dissect the genetic basis of GSL biosynthesis in the seeds of *B. napus* (Li *et al*., 2014; Liu *et al*., 2020; Lu *et al*., 2014; Nour‐Eldin *et al*., 2017; Wang *et al*., 2018). Furthermore, we have gained a significant enhancement for our understanding of GSL formation via the application of associative transcriptomics, and highlighted the importance of orthologues of the MYB transcription factor *HAG1* (Harper *et al*., 2012). More recently, the same gene family was shown to control GSLs content in leaves and a related gene (*HAG3*) was discovered to control GSL content in roots (Kittipol *et al*., 2019).

Although a draft genome sequence of vegetable *B. juncea* was published years ago (Yang *et al*., 2016), and an Indian oilseed *B. juncea* has just released recently (Paritosh *et al*., 2021), if we are to develop a precise genomics‐informed breeding pipeline, then there is a urgent need to acquire high‐quality premium genomic information, develop global landscapes of different genomic organizations and identify the genetic architecture of important quality traits. Updating of genome assemblies and re‐sequencing of another allopolyploid oilseed *B. napus* provided successful case to show how high‐quality genome assemblies facilitate investigations on genomic evolution, structural variation and genetic loci of important traits (Song *et al*., 2020, 2021). However, to date, the genomic basis of structural variations and GSL biosynthesis in *B. juncea* remains largely elusive. In this study, we aimed to explore the global landscapes of genomic signatures and selection footprints for a diverse panel of vegetable and oilseed landraces and breeding lines in *B*. *juncea* crops. We particularly focused on a key trait of GSLs content oilseed responsible for the quality of canola‐quality oil products in *B*. *juncea*.

We first acquired *de novo* chromosome‐scale genome sequences from a low GSL oilseed‐type accession (AU213) and a high GSL vegetable‐type accession (T84‐66). We did this by integrating toolkits from single‐molecule real‐time sequencing and high‐throughput chromosome conformation capture (Hi‐C) technologies, along with high‐resolution linkage mapping. Structural variations were detected and compared between the sub‐genomes of vegetable and oilseed *B*. *juncea* crops. We also achieved a case study involving functional genomics by anchoring two major controlling loci for GSLs, a factor that determines oil quality on the A02 and A09 chromosomes via scored genomic SNP‐based GWAS (SGS‐GWAS). This study will empower to apply genomic selections for desirable target traits and, therefore, provide valuable genome resources for establishing genomic improvement strategies for *B. juncea* and other *Brassica* species.

## Results

### De novo chromosome‐scale genome assemblies of allopolyploid vegetable and oilseed *B. juncea*

**Figure 1:**
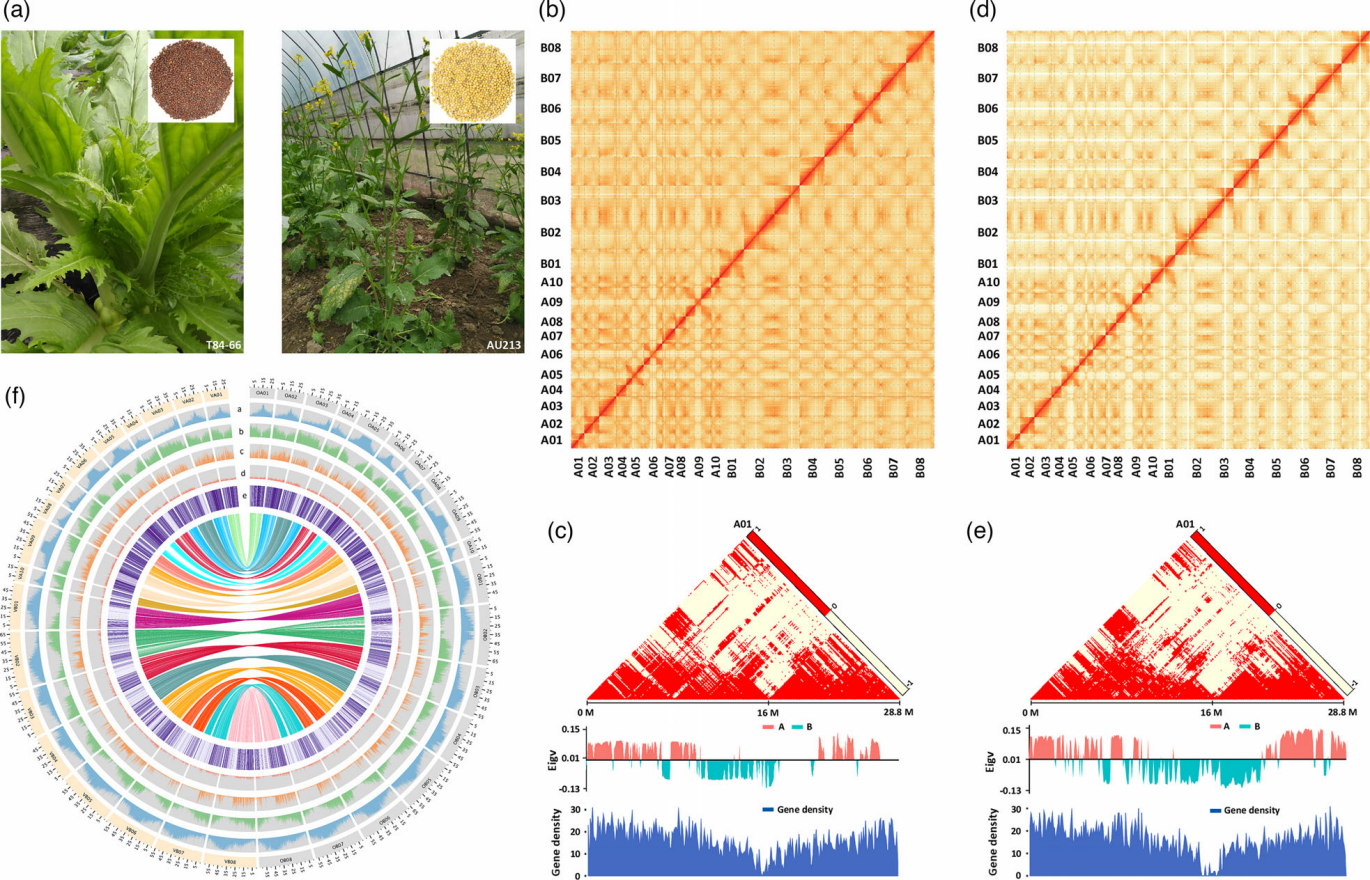
A comparison of genomic signatures between vegetable and oilseed varieties of *B. juncea*. (a) Phenotypes of the ‘double‐high’ vegetable accession (T84‐66) and the Australian ‘double‐low’ oilseed (AU213) accession of *B. juncea*. The T84‐66 plant exhibited swollen stems of the edible organ as vegetables with brown seeds, while the AU213 plant exhibited yellow seeds as oilseeds. (b) Genome‐wide contact matrix of the T84‐66 genome. Colour intensity represents the frequency of contact between the two 500 Kb loci. (c) Interaction frequency, A/B compartment and gene density in the A01 chromosome of T84‐66. (d) Genome‐wide contact matrix for the AU213 genome. The colour intensity represents the frequency of contact between the two 500 Kb loci. (e) Interaction frequency, A/B compartment and gene density in the A01 chromosome of AU213. The colour scale represents the Pearson’s correlation coefficient for the normalized interaction matrix. Eigv, eigenvector value of correlation matrix. (f) Circos plot showing the multidimensional topography of the T84‐66 and AU213 *B. juncea* genomes. A‐E, Concentric circles from outermost to innermost, quantifying the density of repeats (a), gene density (b), SNP density (c), InDel density and (d) PAV distribution. All data are shown in 1 Mb windows sliding 100 kb, and the inner lines show syntenic blocks between T84‐66 (V2) and AU213.

To accomplish precise genomic information from two distinctive *B. juncea* subgroups, vegetable (T84‐66; Figure 1a) and oilseed (AU213; Figure 1a) accessions of *B. juncea* were selected for whole‐genome sequencing. In total, we generated about 146 Gb and 147 Gb of Illumina paired‐end reads for T84‐66 and AU213, respectively; these were then used to estimate the size and assembly of two genomes (Table S1). *K*‐mer analysis revealed that the two genomes were 968 Mb and 938 Mb in size, respectively, with high numbers of repeats and complex genome characteristics (Figure S1). Next, we acquired 251 Gb and 205 Gb PacBio clean subreads with an estimated coverage depth of over 200× for each genome (Tables S2 and S3). Next, we used the Canu pipeline to assembly the subreads into contigs and the assemblies were corrected using the Illumina paired‐end reads.

To construct scaffolds on chromosome scale, we applied over 200× high‐throughput chromosome conformation capture (Hi‐C) datasets for each variety to cluster, order and orient these assemblies (Tables S4 and S5). The Hi‐C datasets exhibited strong signals on the main diagonal for the chromosomes of T84‐66 (Figure 1b) and AU213 (Figure 1d), thus indicating frequent interactions between adjacent loci. The Hi‐C map of *B. juncea* showed contrasting distributions for euchromatin (A) and heterochromatin (B) (Figure 1c and e), in which a similar pattern has already been reported for *B. napus* (Song *et al*., 2020), with relatively lower gene expression pattern in the heterochromatin status near the centromere. Our fine chromosome assemblies exhibited contig N50 with 3.36 Mb (1,151 contigs) for T84‐66 and 4.40 Mb (1,053 contigs) for AU213 (Table 1). These assemblies included 975 and 947 scaffolds for T84‐66 and AU213, respectively, in which the largest scaffolds represented near‐complete chromosome sequences (Table 1 and Table S6).

**Table 1:** Summary of genome assembly and annotation for *B. juncea*

Genomic feature	Vegetable (T84‐66)	Oilseed (AU213)
Total length of contigs	904,836,171	894,631,344
Total length of assemblies	904,876,571	894,664,144
Percentage of anchoring (%)	98.0	98.99
Percentage of anchoring and ordering (%)	90.67	91.76
Number of contigs	1,151	1,053
Contigs N50 (bp)	3,335,154	4,404,270
Contig max (bp)	23,748,072	27,993,914
Number of scaffolds	747	725
Scaffold L50 (bp)	54,976,184	58,712,553
GC content	38.12	37.78
Percentage of repeat sequences	56.53%	57.87%
Number of genes	100,829	100,048

The completeness of the assembly in genic regions was evaluated by the identification of 1407 (97.7%) of the highly conserved core proteins in the BUSCO datasets (V2) for T84‐66 and 1415 (98.3%) for AU213 (Table S7). We also verified 456 (99.6%) and 457 (99.8%) of the 458 CEGs in the CEGMA datasets (V2.5) for these two accessions (Table S8). The accuracy of assembly was supported by alignment of the Illumina short reads, which showed that 97.12% and 96.18% of the reads were mapped to the assemblies for both varieties (Table S9). We also used genome‐ordered graphical genotypes (GOGGs) pipeline (He and Bancroft, 2018) to further validate the assemblies (Figures S2 and S3). In addition, compared with the draft genome of the *B. juncea* vegetable crop that was published previously (T84‐66) (Yang *et al*., 2016), our two new genome sequences represented remarkable improvements in terms of contiguities (Figures S4 and S5; Table S6). Although gap‐filling and high complex regional assembly could be used to further strengthen and improve our assemblies, the current two genomes can serve as better references for studies involving comparative and functional genomics between allopolyploid *B. juncea* crops producing oil and vegetables.

In the assemblies, a total of 512 Mb and 518 Mb of repeated sequences, accounting for 56.5% and 57.9% of the total genome, were annotated for the T84‐66 and AU213 genomes respectively (Table 1; Table S10). Long terminal repeat (LTR)/Gypsy‐type repeat elements were the most abundant, representing 20.9% and 20.2% of the repeated sequences in T84‐66 and AU213, respectively; the second most common element was LTR/Copia (Table S10). These findings were similar to those published for other *Brassica* genomes, including *B. napus, B. rapa* and *B. oleracea* genomes (Liu *et al*., 2014; Song *et al*., 2020).

We predicted that 100,829 and 100,048 genes are present in the T84‐66 and AU213 genomes respectively (Table 1; Tables S11 and S12). In total, 95,966 (95.2%) and 96,588 (96.5%) of the predicted genes were supported by homolog and RNA‐seq predictions, thus showing that our predictions were of high fidelity (Figure S6). Furthermore, 98.2% and 98.4% of the predicted genes were functionally annotated in nr (NCBI) and other datasets (Table S13) in the corresponding T84‐66 and AU213 assemblies. By comparing with the published version of T84‐66 (V1) (Yang *et al*., 2016), we managed to identify an additional 20,779 and 19,880 genes in the genomes vegetable and oilseed plants. Pan‐genome analysis further showed that the approximate gene numbers in our two genomes were similar with *B. napus* (94,586–100,919 genes) (Song *et al*., 2020), thus supporting the BUSCO estimations and the integrity enhancement of the genome assemblies (Table S7). Furthermore, we ensured that over 91.4% and 93.9% of the annotated genes from the T84‐66 and AU213 genomes were supported by other forms of validation including multiple‐blast and Oxford Nanopore Technologies (ONT) long‐reads mapping approaches (Table S14; Dataset S1). In total, 91.9% and 90.6% of unmapped genes from the T84‐66 and AU213 genomes were shown to be homologous with genes from the *Brassicaceae* (Table S14‐S16). These results provided further confirmation of the gene annotations in the new assemblies of T84‐66 and AU213. We also predicted noncoding RNAs in the two‐genome sequences (Table S17). In addition, we annotated 5,764 and 5,788 pseudo‐genes in the two genomes (Table S18). Finally, we charted the genomic annotations of the T84‐66 and AU213 accessions of the *B. juncea* genomes using Circos mapping (Figure 1f).

### Genomic landscapes of structural variations between the vegetable and oilseed of *B. juncea*

**Figure 2:**
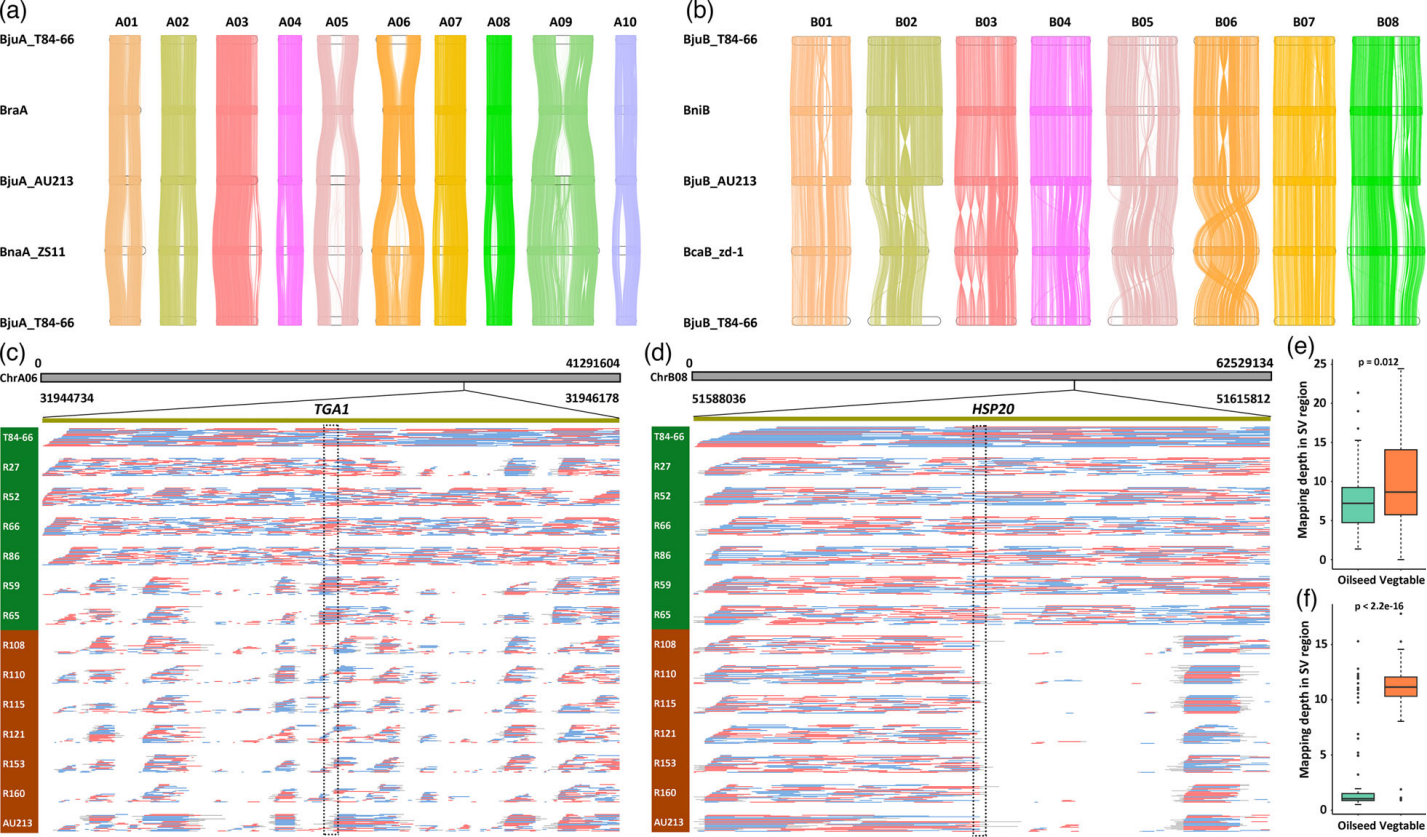
Characterization of genomic variations between vegetable and oilseed variations in *B. juncea*. (a) Genome alignment of the A sub‐genomes in *Brassica* crops. Lines between chromosomes show syntenic regions. (b) Genome alignment of the B sub‐genomes in *Brassica* crops. Lines between chromosomes show syntenic regions. (c) Genotyping of deletions on the A06 using ONT re‐sequencing long reads from 12 varieties of *B. juncea*. *TGA1* was marked in dotted line frame associated with the SVs. (d) Genotyping of deletions on the B08 using ONT re‐sequencing long reads from 12 varieties of *B. juncea*. *HSP20* was marked in dotted line frame associated with the SVs. Varieties in blue background represented vegetables, and varieties in orange background mean oilseeds. (e) Mapping depth in the SV region on the A06 using short reads from a 183 re‐sequencing panel of *B. juncea*. (e) Mapping depth in the SV region on the B08 using short reads from a 183 re‐sequencing panel of *B. juncea*.

Previous research reported that the evolution of *B. juncea* crop most likely underwent specific divergence at some stage leading to two varieties that could be used for two very different applications (vegetable and oilseed) (Yang *et al*., 2016). A similar pattern of evolution has been described to account for the divergence of cotton fibres (Hu *et al*., 2019; Wang *et al*., 2019). Reference‐grade genome assemblies of allopolyploid plants enabled researchers to explore the differentiation in genome organization between sub‐genomes and their diploid ancestors, thus helping us to unravel the possible effects of polyploidization on the genome evolution of sub‐varieties with differential agricultural application (Wang *et al*., 2019; Yang *et al*., 2019). Strikingly, we observed the ubiquitous genomic variations among the A sub‐genomes of *B. juncea*, the A sub‐genome of *B. rapa* and the A sub‐genome of *B. napus* (Figure 2a), and the B sub‐genomes of *B. juncea*, the B genome of *B. nigra* and the B sub‐genome of *B. carinata* (Figure 2b) using a one‐to‐one syntenic block method in *Brassica* species. This finding implicates the multiple events of chromosome recombination and exchange in sub‐genomes during polyploidy speciation and evolution. Furthermore, we also identified structural variations (SVs) in chromosomes A01, A06, A09, B02 and B05 among the sub‐genomes of the new assemblies of A and B sub‐genomes of T84‐66 and AU213 *B. juncea* when compared with newly published oilseed *B. juncea* (Paritosh *et al*., 2021) (Figure S7). Subsequently, we used the long reads of T84‐66 and AU213 to map the newly published oilseed *B. juncea*, thus randomly confirming the SVs in chromosome A01 (Dataset S2).

To better understand genomic divergence between the T84‐66 (vegetable) and AU213 (oilseed) genomes, comparative genomic analysis was applied to distinguish the synteny between the A and B sub‐genomes of the two new assemblies (Figure S8; Figure S9); this revealed substantial genomic variations likewise. We systematically identified genome‐wide single nucleotide polymorphisms (SNPs), insertions/deletions (InDels) and presence/absence variations (PAVs) in the A and B sub‐genomes of T84‐66 and AU213. In total, we anchored 553,697 SNPs (0.96 per kb in average in the A sub‐genome) and 511,918 SNPs (1.64 per kb in average in the B sub‐genome) in T84‐66 and AU213, thus eliminating SNPs in the A sub‐genome mapped to the B sub‐genome, and vice versa (Tables S19 and S20). In addition, we found 352,587 and 336,664 InDels in the A and B sub‐genomes of T84‐66 and AU213 (an average of 0.61 and 1.08 per kb, respectively) (Tables S21 and S22). Furthermore, 24,768 PAVs (> 100 bp) were identified in the A and B sub‐genomes between T84‐66 and AU213 (Tables S23 and S24), in which 3,634 of these PAVs caused variations in 6,425 genes. We randomly selected several PAVs and used PCR to assure the fidelity of these PAVs (Figure S10; Table S25). Some of these genomic variations were located within the genic regions and were predicted to affect gene functions involving biotic and abiotic stresses in the T84‐66 and AU213 crops (Tables S23 and 24). Using PAVs‐based GWAS analysis, causal genetic variations were directly identified for silique length, seed weight and flowering time in *B. napus*, thus demonstrating that PAV‐GWAS was complementary to SNP‐GWAS in associating genotypes with traits (Song *et al*., 2020). Finally, we displayed the panoramic landscapes of genomic variations, including PAVs, SNPs and Indels, between T84‐66 and AU213 allopolyploid *B. juncea* genomes by using a Circos map (Figure 1f).

The high‐quality assembled genomes of the *B. juncea* allopolyploids allowed to identify large structural variations by direct comparative genomic analysis of the two varieties. We observed large inversions between T84‐66 and AU213 in chromosomes A03, A05, A07 and A09 of the A sub‐genome, and chromosomes B01, B02, B04, B05 and B07 of the B sub‐genome. We also identified a large fragment loss in chromosome A06 when aligning the entire genomes (Figure S8). Next, we used Hi‐C data from AU213 to map Hi‐C data from AU213 and T84‐66 to confirm the inversion (12279503‐24970675 bp) in chromosome A05 (Figure S8) and the large fragment loss (13 Mb, 14807362‐27520568 bp) in chromosome A06 (Figure S8). In parallel, we used the single‐molecule real‐time (SMRT) long reads from T84‐66 and AU213 and the ONT re‐sequencing long reads from the 12 varieties of *B. juncea* to map the T84‐66 assembly to validate the large SVs in A05 and A06 (Tables S26 and S27). Results confirmed that the large SVs in A05 and A06 in some accession sequences of the 12 selected varieties, although these SVs were not specific to vegetable and oilseed *B. juncea* crops (Dataset S3), which necessitates further Pan‐genome solutions to resolve their associations with phenotypic traits by introducing accession panels of diverse varieties. In conclusion, the validation datasets disclosed the probable ubiquitous occurrence of large sequences inversion and losses in *B. juncea* genomes during domestication, diversification and breeding processes.

To decipher the SVs‐derived function divergence between vegetable and oilseed varieties of *B. juncea* genomes, we acquired the SVs from the re‐sequenced genomes of 12 selected varieties from the two subgroups of *B. juncea* covering 20× genome using Oxford Nanopore Technology (ONT) long‐reads sequencing (Tables S28). A total of 1354 SVs were uncovered in the two new assemblies including deletions (DEL, 754), duplications (DUP, 15), insertions (INS, 497), inversions (INV, 14) and translocations (TRA, 254). We annotated the genes from 1 kb regions of upstream and downstream of these SVs (Tables S29‐S33). We further confirmed two unique SVs (deletions) in ChrA06 and ChrB08, in which genotyping analysis revealed that these sorted deletions showed conserved variations between the vegetable and oilseed varieties of *B. juncea* genomes (Figure 2c,d). Furthermore, the deletions in ChrA06 and ChrB08 were supported by the short‐reads mapping from a re‐sequencing panel of oilseed and vegetable *B. juncea* (Figure 2e,f). Two significant genetic loci, *TGA1* (TGACG‐BINDING FACTOR 1, *BjuVA06G31030*) and *HSP20* (Heat Shock Protein 20, *BjuVB08G46080*) in ChrA06 and ChrB08, were discovered to be associated with the SVs between the vegetable and oilseed varieties of *B. juncea* genomes (Figure 2c,d). The TGA1 is defined as the immune‐related redox switch depending on salicylic acid pathway (Li and Loake, 2020), and HSP20 is an ubiquitous protein involved in temperature stresses (Waters and Vierling, 2020). We proposed that the *TGA1* and *HSP20* stemming from the SVs are candidate genetic loci associated with natural variations in responses to biotic and abiotic stresses between the vegetable and oilseed varieties of *B. juncea*. These genetic loci are proposed to enlarge their genetic background with biotic and abiotic stress tolerance using reciprocal crossing between vegetable and oilseed varieties of *B. juncea*.

In addition, we found that the frequencies of variations in the B sub‐genome were significantly higher than that in the A sub‐genome, thus indicating that the B sub‐genome underwent higher selective pressures than the A sub‐genome, as supported by higher *Fst* and *π* estimations in the B sub‐genome when compared to the A sub‐genome (Table S34; Figure S11).

### The genetic loci and genomic basis of variations positioning for GSLs biosynthesis in allopolyploid *B. juncea*

To evaluate the genetic basis responsible for the accumulation of GSLs, we then analysed the GSLs in a genetic diversity panel consisting of 183 vegetable and oilseed *B. juncea* accessions. The results showed that sinigrin, an aliphatic GSLs, was the most dominant component in *B. juncea* (Figure S12 and Table S35). Next, we re‐sequenced the diversity panel at 10× coverage and scored genomic SNPs using the T84‐66 genome sequence as a reference for read mapping (Table S36). We, firstly, called a total of 3,495,995 single nucleotide polymorphisms (SNPs) from the re‐sequenced population. As an allotetraploid form of *Brassica*, molecular markers cannot map specifically across the two sub‐genomes (Bancroft *et al*., 2011; Trick *et al*., 2009). To solve this weakness, we filtered the scored SNPs to retain only those that would provide the most reliable mapping, thus retaining only ‘simple’ SNPs (i.e. polymorphisms between resolved bases) in the gene space. Under these stringent filtering rules, a total of 1,508,758 SNPs were scored across the panel. To further reduce noise, we exclude SNPs that lay outside gene spaces. The resulting filtered 689,411 scored SNPs were retained to conduct genotyping on the 183 accessions sequences.

**Figure 3:**
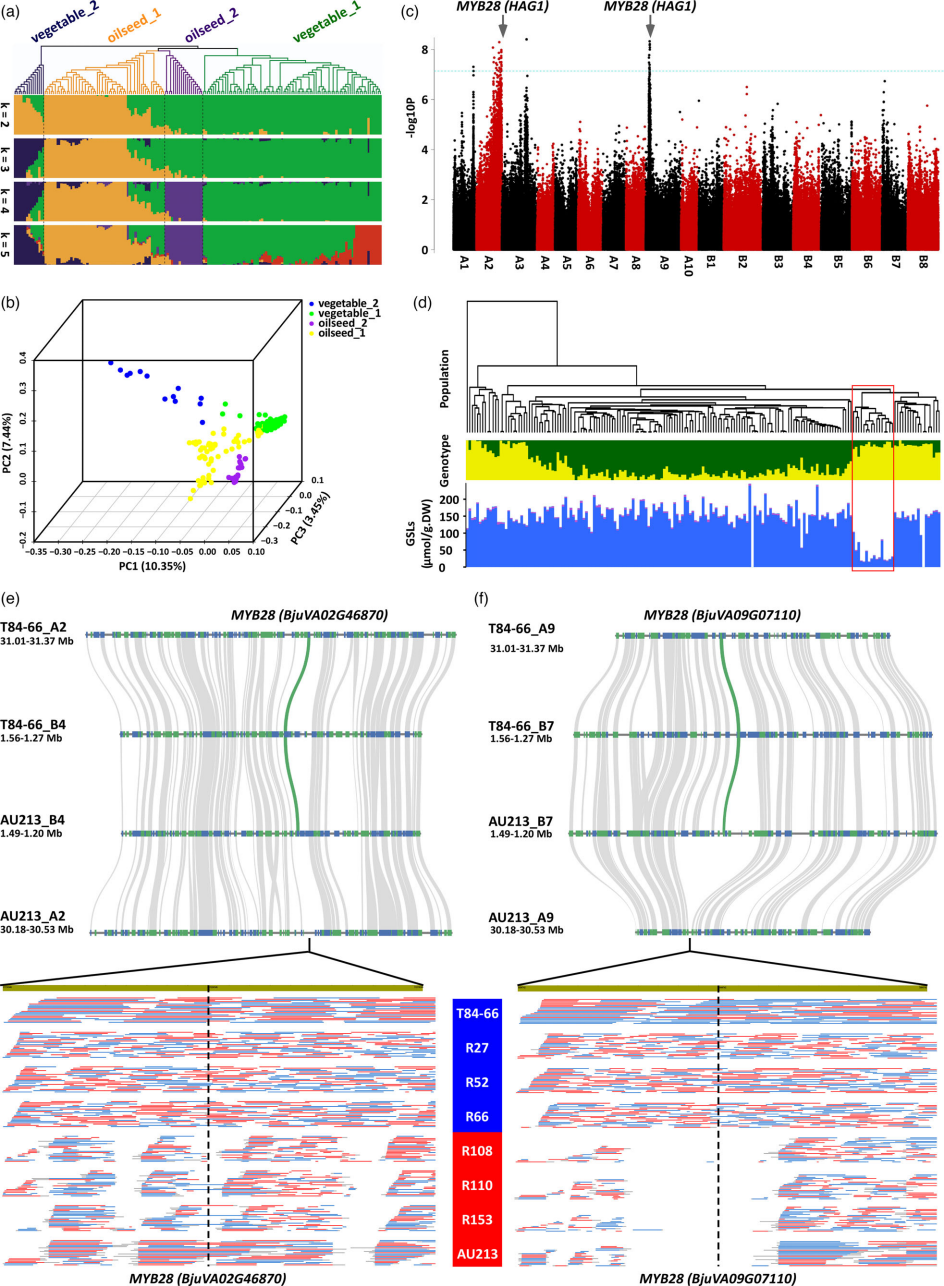
Genetic loci and genomic variations associated with the control of GSLs content in seeds. (a) Phylogenetic tree and population structure of the re‐sequenced *B. juncea* accession panel. (b) Principal component analysis of the re‐sequenced *B. juncea* accession panel. (c) Manhattan plot for SNP markers ordered by position in the genome (x axis) that are associated with variations for GSLs content in seed. The blue dashed line represents the 0.05 significance threshold after adjustment for false discovery rate. (d) Associated analyses of GSLs content and SNP variations in the re‐sequenced *B. juncea* accession panel. The red frame shows accessions with low levels of GSLs. (e) Copy number variation of *MYB28* (*BjuVA02G46870*) in T84‐66 and AU213 genomes and ONT long‐reads mapping from varieties with low and high GSLs accumulation. (f) Copy number variation of *MYB28* (*BjuVA09G07110*) in T84‐66 and AU213 genomes and ONT long‐reads mapping from varieties with low and high GSLs accumulation.

Secondly, we used these SNPs to create a neighbour‐joining phylogenetic tree (Figure S13). By integration analysis of population structure (Figure 3a), principal component analysis (Figure 3b), agricultural usage and geographical distributions (Figure 3a and b), these accessions were clustered into four main subgroups: vegetable_1, vegetable_2, oilseed_1 and oilseed_2 subgroups. The vegetable_1 group included leafy and stem types, and the vegetable_2 group consisted of root and leafy types that are only derived and used for vegetables in China. The oilseed_1 group contained oilseed types that are used for the production of oil in China, Europe and Australia. The oilseed_2 group contained oilseed types that are used for oils production in India. These four subgroup classifications were strongly supported by previous studies of phylogenomic evolution using a specific locus amplified fragment sequencing (Yang *et al*., 2018) and simple sequence repeat (SSR) markers (Chen *et al*., 2013), although two lineages including seven subgroups were suggested by another set of nuclear and chloroplast SSR markers (Kaur *et al*., 2014). Relative kinship analysis further indicated that most accessions in the panel had very weak levels of kinship, probably due to the broad collections of genotypes presented, and these accessions could, therefore, be used for GWAS analysis (Figure S14). The linkage disequilibrium (LD) decay and relative higher LD distance in the oilseed_2 subgroup (from India) indicated intensive selections on the genome than on other subgroups in accordance with the practical records of breeding history as main oil crop in India (Figure S15).

Next, we undertook a genome‐wide association study (GWAS) to evaluate the total GSLs content in seeds. Our results demonstrated major associations in two regions of chromosomes A2 and A9 on the genome. Both regions contain orthologues of *HAG1* (*MYB28*), *BjuVA02G46870.1* and *BjuVA09G07110*.*1*, major regulatory genes that control the biosynthesis of aliphatic GSLs in seed (Figure 3c; Table S37). The targeted silencing of *MYB28*, homolog of *BjuVA02G46870.1*, once confirmed the role of this gene in the down‐regulating GSLs synthesis in the oilseed *B. juncea* (Augustine *et al*., 2013). The associated peak on A2 was particularly broader, thus suggesting its recent introgressions into the germplasm there having been insufficient generations to allow recombination to reduce its size in breeding history. In this study, we found two genetic loci involved in GSL variations in the panel collection of *B. jucnea* accessions, in A02 and A09, in which the MYB28 in A09 is firstly reported to be associated with the accumulation of GSL in *B. jucnea*. We would like to emphasize that the identification of control of seed GSL content by *MYB28* orthologues is novel for *B. juncea* crop and that these findings could not be inferred from our understanding of *B. napus*. We also found that the accessions with low levels of GSLs are associated with the observed variations in SNPs in our panel accessions (Figure 3d), which confirmed the genetic causality of *MYB28* for low GLSs accumulation. Interestingly, we found that the variations on *MYB28* loci were caused by copy number variations (CNVs) between T84‐66 and AU213 accessions, in which one copy was lost both on A2 and A9 chromosomes of AU213 (Figure 3e and f). Furthermore, the CNVs on *MYB28* were validated by ONT long‐reads mapping between accessions with low and high GSLs (Figure 3e and f). A 4827 bp deletion covered the *MYB28* (*BjuVA02G46870.1*) that was identified in AU213, although it seems that the ONT long reads overlapped on *MYB28* (*BjuVA02G46870.1*) (Figure S16). These results highlight a promising and precise strategy for breeding oilseed crops containing favourably low levels of GSLs oilseed crops by targeting the genetic loci identified in this study.

## Discussion

*Brassica juncea* is an allopolyploid crop species with two contrasting types, as leafy vegetables and oilseed crops. In the present study, we achieved premium quality chromosome‐scale genome sequences for both types of crop. Compared with previously published draft genomes of vegetable *B. juncea* (T84‐66, V1) (Yang *et al*., 2016), the current two new genomes represent significant improvement in terms of contiguity with their organizations being validated using GOGGs. There are numerous structural variations in sub‐genomes A and B between vegetable and oilseed *Brassica juncea* and their ancestral *Brassica rapa* (AA) and *Brassica nigra* (BB), implicating extensive sub‐genomes recombination and rearrangement during polyploidy genome evolution. The comparative genomic results partially explain the genome evolution and diversity in *Brassica juncea* crops with wide adaptability and climate resilience. Our accomplishments should enable the research community to undertake elaborate comparative and functional genomics studies in polyploidy *B. juncea* for varied purposes.

These high‐quality genome assemblies can also be used to dissect the genetic basis for many key traits of agronomic importance. In the present study, we undertook GWAS analysis to investigate GSLs content, an important trait for oil quality. The genetic diversity panel assembled and genotyped by genome re‐sequencing included accessions that showed remarkable differences in GSLs contents of seeds. Accessions with a lower content of GSLs were developed to meet ‘Canola’ quality standards (low levels of erucic acid in oil and low levels of GSLs in residual meal). Our SGS‐GWAS analysis enabled the identification of two major control loci, each containing orthologues of MYB28 (*HAG1*), a transcription factor known to control the biosynthesis of aliphatic GSLs as the major compounds in the seeds of both *B. juncea* and *B. napus* (Hirai *et al*., 2007). One of the loci is in common on chromosome A9 with *B. napus*, but the other on A2 differs, being homoeologous to the second locus detected in *B. napus,* which was on chromosome C2. An important goal is for breeders to develop *Brassica* oilseed cultivars with desirable ‘double‐low’ of GSLs and erucic acid as this would address issues related to anti‐nutritional, oncogenic and toxicity effects. In view of the agricultural and economic significance of oilseed *Brassica* crops, it is crucial to dissect the genetic basis that underlies the accumulation of GSLs. In particular, it is important to identify more favourable alleles or target genes that might allow breeders to create advanced cultivars with ‘Canola’ quality.

As well as being unique to *B. juncea*, the chromosome A2 association peak was exceptionally broader, encompassing approximately half of the chromosome. This lack of a breakdown in the linkage is indicative of relatively shorter generations since the introduction of the variant into the populations during a history of breeding with low GSLs accessions. The pioneering work on low GSLs *B. juncea* breeding was developed from an interspecific cross between an Indian oilseed *B. juncea* line with a low GSLs *B. rapa*, followed by one backcrossing to an Indian oilseed *B. juncea* line and the selfing of backcrossed plants (Love *et al*., 1990). However, no associations were detected in the B genome, thus demonstrating that sub‐genome A rather than B was prone to genomic variations under selection with regards to the accumulation of GSLs. Our present data will empower the structural variations studies in polyploidy genome evolution and the genomic selections of crops with favourable levels of GSL biosynthesis, and will help us to develop genomics‐assisted portfolios for designing the ‘canola’ quality of *B. juncea* oilseed crop and other species of oilseed *Brassica*.

### Online content

Methods, additional references, source data, supplementary information and acknowledgements; details of author contributions and competing interests and any associated codes are available online.

## Experimental procedures

### Genome‐wide association scanning

Genotyping and SNP calling were performed using a pipeline that employed a suite of bioinformatic tools. BWA (Version: 0.7.17‐r1188) was used to map Illumina paired‐end DNA sequencing reads of 183 accessions to the assembled *B. juncea* (T84‐66) genome using the default parameters. SAMtools (Version: 1.10) was used to index and pileup mapping results. SNP detection was performed using the VarScan (v2.4.3). VarScan mpileup2snp was used with min‐var‐freq set to 0.01 and min‐coverage set to 7. Then, a custom R script was used to process SNPs with the following criteria: missing data exclusion threshold set to 25%, only two alleles allowed, merely simple SNPs retained and SNPs eliminated for invariant across lines (only one variant detected). Under these stringent filtering, a total of 1,508,758 SNPs were scored across the panel. To further reduce noise, an R script was used to exclude SNPs outside gene spaces.

GWAS analyses were performed using the programs and parameters essentially as described (Harper *et al*., 2020). The SNP dataset was entered into the program PSIKO (Popescu *et al*., 2014) to assess the population structure and produce a Q matrix, which was composed of two population clusters. The SNP genotypes, Q matrix and trait scores for 183 accessions were incorporated into a compressed mixed linear model (Zhang *et al*., 2010) implemented in the GAPIT R package (Lipka *et al*., 2012), with missing data imputed to the major allele. The kinship matrix used in this analysis was also generated by GAPIT. Manhattan plots were plotted by R script, which displayed the results of association with individual trait. The 5% significance false discovery rate was calculated and displayed.

## Author contributions

M.Z. and J.Y. coordinated the project, conceived and designed the experiments. J.Y., J.W. and X.L. assembled and performed bioinformatics analyses of the genomes. Z.L., L.Z., T.S., X.L. and Z.H. prepared samples for sequencing and analysed data. S.C., Y.G. and Z.L. provided oilseed *B. juncea* accessions and discussed the draft. H.H. analysed glucosinolates components. I.B. and Z.H. conducted and interpreted the GWAS analysis. J.Y. wrote the manuscript. M.Z. and I.B. revised the manuscript.

## Competing interests

The authors declare no competing interests.

## Supporting information


**Figure S1** Estimation of genome size, heterozygosity and repeated sequences percentage using K‐mer analysis of Illumina sequencing data.
**Figure S2** Genome sequence organization of vegetable *B. juncea* (T84‐66) with assessment using genome‐ordered graphical genotypes and chromosome A01 as examples.
**Figure S3** Genome sequence organization of oilseed *B. juncea* (AU213) with assessment using genome‐ordered graphical genotypes and chromosome A01 as examples.
**Figure S4** Synteny analysis between new assembly of vegetable (T84‐66, V2) and published assembly of V1.0 (T84‐66, V1).
**Figure S5** Synteny analysis between new assembly of vegetable (AU213) and published assembly of V1.0 (T84‐66, V1).
**Figure S6** Summary of gene annotation by RNA‐seq and homological genes.
**Figure S7** Synteny analysis between new assembly of A sub‐genomes of vegetable (T84‐66) and oilseed (AU213) *B. juncea* with a newly published oilseed *B. juncea* (2020).
**Figure S8** Synteny analysis between new assembly of A sub‐genomes of vegetable (T84‐66) and oilseed (AU213) *B. juncea*.
**Figure S9** Synteny analysis between new assembly of B sub‐genomes of vegetable (T84‐66) and oilseed (AU213) *B. juncea*.
**Figure S10** Validation of selected PAVs in T84‐66 and AU213 using PCR amplification.
**Figure S11** *Fst* and *π* estimations of A and B sub‐genomes using re‐sequencing population of *B. juncea*.
**Figure S12** Glucosinolates (GSLs) components analysis using HPLC in the re‐sequencing population of *B. juncea*.
**Figure S13** A phylogenetic neighbour‐joining tree constructed from 183 accessions of *B. juncea*.
**Figure S14** Relative kinship analysis in the re‐sequencing population of *B. juncea*.
**Figure S15** LD decay distance in the re‐sequencing population of *B. juncea*.
**Figure S16** A 4827 bp deletion covered the *MYB28* (*BjuVA02G46870.1*) in AU213.
**Table S1** Summary of Illumina clean reads for two accessions of allopolyploid *B. juncea*.
**Table S2** Summary of PacBio clean subreads for two accessions of allopolyploid *B. juncea*.
**Table S3** Summary of subreads length distributions for two accessions of allopolyploid *B. juncea*.
**Table S4** Summary of Hi‐C reads mapping.
**Table S5** Summary of assembly by using Hi‐C data.
**Table S6** Summary of genome assemblies for *B. juncea*.
**Table S7** BUSCO assessment of *B. juncea* genomes assembly.
**Table S8** Assembly assessment by CEGMA.
**Table S9** Assembly assessment by Illumina‐seq reads.
**Table S10** Summary of repeated sequences annotation.
**Table S11** Summary of gene prediction.
**Table S12** Characterization of genes in vegetable and oilseed of B. *juncea*.
**Table S13** Summary of gene annotation by different databases.
**Table S14** Summary of annotated genes of T84‐66 and AU213 validated by multiple‐blast and long‐reads mapping.
**Table S15** Annotation of unmapped genes from T84‐66 genome.
**Table S16** Annotation of unmapped genes from AU213 genome.
**Table S17** Summary of noncoding RNA predication.
**Table S18** Summary of pseudo‐gene predication.
**Table S19** SNPs identification in the A sub‐genome of T84‐66 (V2) and AU213.
**Table S20** SNPs identification in the B sub‐genome of T84‐66 (V2) and AU213.
**Table S21** Indels identification in the A sub‐genome of T84‐66 (V2) and AU213.
**Table S22** Indels identification in the B sub‐genome of T84‐66 (V2) and AU213.
**Table S23** PAVs and related genes in between A sub‐genome of T84‐66 (V2) and AU213.
**Table S24** PAVs and related genes in between B sub‐genome of T84‐66 (V2) and AU213.
**Table S25** Primers to confirm PAVs used in this study.
**Table S26** Depth statistics of long reads to A05 of T84‐66 assembly.
**Table S27** Depth statistics of long reads to A06 of T84‐66 assembly.
**Table S28** Samples used in re‐sequencing by ONT method.
**Table S29** Annotation on genes involved in DEL‐type SVs.
**Table S30** Annotation on genes involved in DUP‐type SVs.
**Table S31** Annotation on genes involved in INS‐type SVs.
**Table S32** Annotation on genes involved in INV‐type SVs.
**Table S33** Annotation on genes involved in TRA‐type SVs.
**Table S34** Statistics of variation density between sub‐genomes.
**Table S35** Glucosinolates components in seeds of re‐sequencing population.
**Table S36** Statistics of re‐sequencing of 187 accessions of B. juncea.
**Table S37** Candidate genes associated with glucosinolate accumulation by GWAS.

## Data Availability

The genome assemblies of *B. juncea* varieties T84‐66 and AU213 are deposited in NCBI GenBank under the BioProject accession number PRJNA697823. The raw sequencing data have been deposited in the NCBI Sequence Read Archive under the accession number PRJNA697823.
