# Supplementary material for: Genomic signatures of vegetable and oilseed allopolyploid Brassica juncea and genetic loci controlling the accumulation of glucosinolates
Source: Plant Biotechnol J. 2021 Oct 1;19(12):2619–28. doi: 10.1111/pbi.13687 (PMC8633494; doi:10.1111/pbi.13687)
Supplement: Supplementary file 1 — Figure S1 Estimation of genome size, heterozygosity and repeated sequences percentage using K‐mer analysis of Illumina sequencing data. Figure S2 Genome sequence organization of vegetable B. juncea (T84‐66) with assessment using genome‐ordered graphical genotypes and chromosome A01 as examples. Figure S3 Genome sequence organization of oilseed B. juncea (AU213) with assessment using genome‐ordered graphical genotypes and chromosome A01 as examples. Figure S4 Synteny analysis between new assembly of vegetable (T84‐66, V2) and published assembly of V1.0 (T84‐66, V1). Figure S5 Synteny analysis between new assembly of vegetable (AU213) and published assembly of V1.0 (T84‐66, V1). Figure S6 Summary of gene annotation by RNA‐seq and homological genes. Figure S7 Synteny analysis between new assembly of A sub‐genomes of vegetable (T84‐66) and oilseed (AU213) B. juncea with a newly published oilseed B. juncea (2020). Figure S8 Synteny analysis between new assembly of A sub‐genomes of vegetable (T84‐66) and oilseed (AU213) B. juncea. Figure S9 Synteny analysis between new assembly of B sub‐genomes of vegetable (T84‐66) and oilseed (AU213) B. juncea. Figure S10 Validation of selected PAVs in T84‐66 and AU213 using PCR amplification. Figure S11 Fst and π estimations of A and B sub‐genomes using re‐sequencing population of B. juncea. Figure S12 Glucosinolates (GSLs) components analysis using HPLC in the re‐sequencing population of B. juncea. Figure S13 A phylogenetic neighbour‐joining tree constructed from 183 accessions of B. juncea. Figure S14 Relative kinship analysis in the re‐sequencing population of B. juncea. Figure S15 LD decay distance in the re‐sequencing population of B. juncea. Figure S16 A 4827 bp deletion covered the MYB28 (BjuVA02G46870.1) in AU213. Table S1 Summary of Illumina clean reads for two accessions of allopolyploid B. juncea. Table S2 Summary of PacBio clean subreads for two accessions of allopolyploid B. juncea. Table S3 Summary of subreads length dist [file PBI-19-2619-s001.zip › pbi13687-sup-0001-Supinfo.pdf]

Methods

DNA extraction and genome sequencing. Genomic DNAs of T84-66 and AU213 leaves were extracted by a modified CTAB method[1], in which the CTAB extraction buffer contains 0.1 M Tris-HCl, 0.02 M EDTA, 1.4 M NaCl, 3% (w/v) CTAB, 5% (w/v) PVP K40 and 2% β-mercaptoethanol. Genomic DNAs were prepared for libraries construction and sequencing.

For PacBio sequencing, genomic DNAs were broken into 20 kb sized fragments. and purified and concentrated with AmpureXP beads (Agencourt), and treated with end-repair, adapter ligation and exonuclease digestion by Single-Molecule Real Time (SMRT) manufacturer's protocol. After size selection and isolation, fractions were purified using AmpureXP beads (Beckman Coulter, Inc.). Single-molecule sequencing was done on a PacBio RSII system after removing subreads that shorter than 500bp.

For Hi-C sequencing, a library with fragments ranging from 300 to 700 bp was constructed. Genomic DNAs were treated with *HindIII* enzyme after cross-linked to get digested fragments with sticky ends which were then biotinylated and ligated to form chimeric circles, and sequenced at Illumina Hiseq Xten platforms. Adapter sequences of raw reads were trimmed with cutadapt v1.0[2].

Regarding to re-sequencing using ONT platform, the purity, concentration and completeness of total DNA were qualified by Nanodrop, Qubit and 0.35% agarose gel electrophoresis. Then, total DNAs were fragmented by gTube into sequences with average length of 8kb. Nanopore libraries were constructed after DNA repair and adapter ligation using a Ligation Sequencing Kit (SQK-LSK109) and quantitative

deletion by Qubit. The libraries were subjected to be sequenced on the PromethION sequencer (Oxford Nanopore Technologies, UK). Adapters and low quality, short reads (<500bp) were filtered, and finally the average 24× depth clean data were obtained.

About re-sequencing using Illumina platform, libraries with 270 bp sized fragments were prepared for paired-end (PE) sequencing following the Illumina's protocols (Illumina, USA). The fragmented Genomic DNAs were added an adenine and sequencing adaptors at the 3' end and isolated by ligated. 2 % agarose gels, and then sequenced on an Illumina platform with a paired-end sequencing strategy (2 x 150bp). The contaminated reads were screened by alignment to NCBI-NR database using BWA v0.7.13[3]. The duplicated read pairs were removed by FastUniq v1.1[4], and filtered using the following conditions: (1) reads with ≥10% unidentified nucleotides (N), (2) reads with >10 nucleotides aligned to the adapter, allowing ≤10% mismatches, (3) reads with >50% bases having Phred quality <5.

As RNA-seq using ONT platform, total RNAs were extracted from leaves, roots, buds and stems of AU213 and T84-66 using Tiangen-DP441 kit. RNA quality was detected by RNase free agarose gel electrophoresis. RNA concentration and purity were detected by Nanodrop 2000. Agilent 2100 (Agilent Technologies, Santa Clara, CA, United States) was applied to detect 28S/18S and RIN values. Poly(A) mRNAs were purified from total RNA using mRNA Capture Beads (Novazan) and enriched. Reverse transcription primers were bind to the tail of Poly(A) mRNAs through annealing. Then the first strands were synthesized by RT reverse transcriptase. The double stranded cDNA was amplified by PCR. NEBNext FFPE DNA Repair Mix and NEBNext Ultra II End

Repair/dA-Tailing Module were used for nucleic acid fragment repair and adding adenine to the end of fragments. Sequencing was performed on the Promethlon 48 platform of Oxford NanoPore Technologies after adapter ligation using SQK-LSK109 (ONT) kit. After filtered reads with low quality, clean reads obtained were aligned to the reference genome using Minimap2 ("–ax splice –uf –k14"). Stringtie 2 was used for gene expression with options "-L -G GFF_file -A FPKM_file -e"[5].

***De novo* genome assembly and chromosome construction.** Considering error rate of Pacbio reads, the filtered reads were corrected by Canu pipeline[6] ('corrected Error Rate=0.045') and then prepared for assembly by Falcon v0.3.0 and Wtdgb. The Illumina data were integrated to correct the draft contigs using Pilon v1.22[7], and Hi-C clean reads were then aligned to the assembled contigs with BWA to evaluate the ratio of mapped reads. HiC-Pro v2.10.0[8] were uesd to identify the valid interaction read pairs. The draft contigs were separately broken into fragments with a length of 50 kb, and clustered by LACHESIS[9] using valid interaction read pairs.

Genome assembly assessment and annotation. By mapping 1,440 Benchmarking Universal Single-Copy Orthologs (BUSCOs) and 458 Core Eukaryotic Genes (CEGs) to the assembly genomes, the assembly genomes integrity assessment were checked by BUSCO v3.0.2b[10] and CEGMA v2.5[11]. Additionally, Illumina short reads were also mapped to the reference genome by BWA for assessment. The genome-ordered graphical genotypes pipeline (GOGGs) were used to validate the assembly[12].

For repeats annotation, a *novo* repeat library were constructed by LTR-FINDER[13], MITE-Hunter[14], RepeatScout v1.0.5[15] and PILER-DF[16]. The outputs were merged and classified by PASTEClassifier v1.0[17]. The final library was created by the *de novo* constructed database and the Repbase database v20.01[18], and the repeat sequences were finally identified by RepeatMasker program v4.0.6[19].

For gene prediction and annotation, protein-coding genes were predicted by *de novo*, protein homology and RNA-Seq approaches, in which *de novo* gene prediction were performed by Genscan v1.0[20], Augustus v2.4[21], GlimmerHMM v3.0.4[22], GeneID v1.4 and SNAP packages[23]; protein homology prediction was performed by making alignment of our assemblies to homologous genes from *Arabidopsis thaliana, Brassica napus, Brassica oleracea, Brassica rapa* using GeMoMa v1.3.1[24]; and RNA-Seq data were used for assembly based on reference transcripts and TransDecoder v2.0 by Hisat v2.0.4 and Stringtie v1.2.3[25]. Additionally, GeneMarkS-T v5.1[26] were used for gene prediction. Gene structures were modeled by PASA v2.0.2[27] ,in which the regions of CDS were identified by TransDecoder v3.0.1 and GeneMarkS-T v5.1[26], and then EVidenceModeler v1.1.1[28] was applied to integrate these data. If at least one exon of a gene can align the homolog using position alignment, we decide that the gene is supported by the prediction. By blasting sequences of the predict genes with nucleotide and protein sequence databases, including KOG[29], KEGG[30], NCBI-NR and TrEMBL (*E*-value = 1e-5)[31], gene annotations were completed. Finally, Blast2GO based on NCBI database was used to conduct gene ontology (GO)[32] analysis.

Regarding to validation of gene annotation, we performed the following analyses.

Firstly, we compared annotated genes from T84-66 and AU213 genomes with the genes from previous published genome (2016) using blast, and identified non-matched genes by the identity and coverage over 70%. Then, we verified these no hit genes using following strategies: (1) to compare these no hit genes with transcriptome data from Illumina sequencing to identity hit genes using blast by the identity and coverage over 70%; (2) to find expressed genes in transcriptome data from Oxford Nanopore Technologies long-reads sequencing using Stringtie 2; (3) to compare these no hit genes with annotated gene from the newly published *B. juncea* genome (2020) to identity hit genes using blast by the identity and coverage over 70%; (4) to find orthologue genes from the newly published *B. juncea* genome (2020) using Orthofinder; (5) to find genes located in the non-colinear regions between T84-66/AU213 genomes and the published genome (2016), and mapped the reads from Oxford Nanopore Technologies sequencing to T84-66/AU213 genomes.

For noncoding RNA and pseudogene annotation, Rfam database v32.0[33] was used to identify rRNA by BLAST with the setting of *E-value*=1e-10 and identity > 95%. tRNA genes and miRNAs were predicted by tRNAScan-SE algorithms[34] and INFERNAL v1.1[35], respectively. GenBlast A v1.0.4 (*E-value*=1e-5)[36] and GeneWise v 2.4.1[37] were used to analyze pseudogene sequencesand non-mature termination codes and frame shift mutations separately.

Genome syntenic blocks and chromosomal structure variations. Syntenic gene blocks between A and B subgenomes were analyzed by All-vs-All blastp (E-value =1e-5) and

homologous genes were found by MCScanX[38]. Genome alignment between T84-66 and AU213 was performed by MUMmer 3.23[39], in which SNPs and InDels were identified by running show '–snp' in alignment blocks, and structure variations (inversions and translocations) were extracted by personalized perl script based on MUMmer results. In addition, both inversions and translocations were identified with a length of > 1000 bp and identity of > 90%. PAVs were identified by ppsPCP with the settings '--coverage 0.5 --sim_pav 0.9'[40].

Regarding to structural variation calling, clean reads from ONT resequencing were mapped with the T84-66 genome using Minimap2 (-AX MAP-ONT --MD, other parameters default)[41] to mapping ratios and depth distribution. Sniffles was used for structural variation detection with parameters "--min_support 10, --max_num_splits 7, --max_distance 1000, --min_length 30"[42].

Transcriptome sequencing and identification of differential homoeologs expression. Total RNA was extracted by TRIzol™ Reagent and purified by poly-T oligo-attached magnetic beads, and then selected on the Agilent Bioanalyzer 2100 system. Libraries with 300bp insert size were generated by NEBNext UltraTM RNA Library Prep Kit and then sequenced on the Illumina HiSeq xten platform (Illumina, USA). TopHat was used to bulid the alignment of clean reads to the reference genome, and gene expression was quantified and normalized by Cufflinks in RPKM (reads per million per kilo bases). More than 2-fold change in expression and false discovery rate (FDR) < 0.05 was identified as criteria of differentially expressed genes.

Quantitative analysis of Desulfo-Glucosinolates. Quantification of GSLs in seeds of *B. juncea* was carried out using the HPLC method[43].

Phylogenetic analysis and population structure. The phylogenetic trees were constructed by MEGA X[44] with NJ model using the retained sequences. The population structure was inferred by Admixture v1.22[45]. Genetic clusters (K) was set as 1~10 in advance for clustering, and the clustering results were cross-verified. In addition, principal component analysis (PCA) was performed by EIGENSOFT for mutual authentication with the Admixture[46].

Linkage diseqilibrium analysis. Linkage diseqilibrium (LD), the linkage imbalance between two SNPs over 1000 kb on the same chromosome was calculated by plink2[47]. The curve plots were fitted with SNP distance and linkage imbalance strength, and the heatmaps were plot by LD heatmap package in R.

References

1. Doyle, J.J. & Doyle, J.L. Isolation of plant DNA from fresh tissue. *Focus* **12**, 13-15 (1990).
2. Martin, M. Cutadapt removes adapter sequences from high- throughput sequencing reads. *EMBNet.journal* **17**, 10-12 (2011).
3. Li, H. & Durbin, R. Fast and accurate short read alignment with Burrows-Wheeler transform. *Bioinformatics* **25**, 1754-1760 (2009).
4. Xu, H.B. *et al.* FastUniq: A Fast De Novo Duplicates Removal Tool for Paired Short Reads. *Plos One* **7**(2012).
5. Kovaka, S. *et al.* Transcriptome assembly from long-read RNA-seq alignments with StringTie2. *Genome Biology* **20**(2019).
6. Koren, S. *et al.* Canu: scalable and accurate long-read assembly via adaptive k-mer weighting and repeat separation. *Genome Research* **27**, 722-736 (2017).
7. Walker, B.J. *et al.* Pilon: An Integrated Tool for Comprehensive Microbial Variant Detection and Genome Assembly Improvement. *Plos One* **9**(2014).
8. Servant, N. *et al.* HiC-Pro: an optimized and flexible pipeline for Hi-C data processing. *Genome Biology* **16**(2015).
9. Burton, J.N. *et al.* Chromosome-scale scaffolding of de novo genome assemblies based on chromatin interactions. *Nature Biotechnology* **31**, 1119-+ (2013).
10. Simao, F.A., Waterhouse, R.M., Ioannidis, P., Kriventseva, E.V. & Zdobnov, E.M. BUSCO: assessing genome assembly and annotation completeness with single-copy orthologs. *Bioinformatics* **31**, 3210-3212 (2015).
11. Parra, G., Bradnam, K. & Korf, I. CEGMA: a pipeline to accurately annotate core genes in eukaryotic genomes. *Bioinformatics* **23**, 1061-7 (2007).
12. Yang, J.H., Ji, C.M., Liu, D.Y., Wang, X.W. & Zhang, M.F. Reply to: 'Organization of the genome sequence of the polyploid crop species Brassica juncea'. *Nature Genetics* **50**, 1497-1498 (2018).
13. Xu, Z. & Wang, H. LTR_FINDER: an efficient tool for the prediction of full-length LTR retrotransposons. *Nucleic Acids Research* **35**, W265-W268 (2007).
14. Han, Y.J. & Wessler, S.R. MITE-Hunter: a program for discovering miniature inverted-repeat transposable elements from genomic sequences. *Nucleic Acids Research* **38**(2010).
15. Price, A.L., Jones, N.C. & Pevzner, P.A. De novo identification of repeat families in large genomes. *Bioinformatics* **21**, I351-I358 (2005).
16. Edgar, R.C. & Myers, E.W. PILER: identification and classification of genomic repeats. *Bioinformatics* **21**, I152-I158 (2005).
17. Wicker, T. *et al.* A unified classification system for eukaryotic transposable elements. *Nature Reviews Genetics* **8**, 973-982 (2007).
18. Bao, W.D., Kojima, K.K. & Kohany, O. Repbase Update, a database of repetitive elements in eukaryotic genomes. *Mobile DNA* **6**(2015).
19. Chen, N. Using RepeatMasker to identify repetitive elements in genomic sequences. *Current Protocols in Bioinformatics* **Chapter 4**(2004).
20. Burge, C. & Karlin, S. Prediction of complete gene structures in human genomic DNA. *Journal of Molecular Biology* **268**, 78-94 (1997).
21. Stanke, M. & Waack, S. Gene prediction with a hidden Markov model and a new intron

submodel. *Bioinformatics* **19**, Ii215-Ii225 (2003).
22. Majoros, W.H., Pertea, M. & Salzberg, S.L. TigrScan and GlimmerHMM: two open source ab initio eukaryotic gene-finders. *Bioinformatics* **20**, 2878-2879 (2004).
23. Blanco, E., Parra, G. & Guigó, R. Using geneid to identify genes. *Current Protocols in Bioinformatics* **4.3**, 1-28 (2007).
24. Keilwagen, J. *et al.* Using intron position conservation for homology-based gene prediction. *Nucleic Acids Research* **44**(2016).
25. Pertea, M., Kim, D., Pertea, G.M., Leek, J.T. & Salzberg, S.L. Transcript-level expression analysis of RNA-seq experiments with HISAT, StringTie and Ballgown. *Nature Protocols* **11**, 1650-1667 (2016).
26. Tang, S.Y.Y., Lomsadze, A. & Borodovsky, M. Identification of protein coding regions in RNA transcripts. *Nucleic Acids Research* **43**(2015).
27. Haas, B.J. *et al.* Improving the Arabidopsis genome annotation using maximal transcript alignment assemblies. *Nucleic Acids Research* **31**, 5654-5666 (2003).
28. Haas, B.J. *et al.* Automated eukaryotic gene structure annotation using EVidenceModeler and the program to assemble spliced alignments. *Genome Biology* **9**(2008).
29. Tatusov, R.L. *et al.* The COG database: an updated version includes eukaryotes. *Bmc Bioinformatics* **4**(2003).
30. Kanehisa, M. & Goto, S. KEGG: Kyoto Encyclopedia of Genes and Genomes. *Nucleic Acids Research* **28**, 27-30 (2000).
31. Boeckmann, B. *et al.* The SWISS-PROT protein knowledgebase and its supplement TrEMBL in 2003. *Nucleic Acids Research* **31**, 365-370 (2003).
32. Conesa, A. *et al.* Blast2GO: a universal tool for annotation, visualization and analysis in functional genomics research. *Bioinformatics* **21**, 3674-3676 (2005).
33. Griffiths-Jones, S. *et al.* Rfam: annotating non-coding RNAs in complete genomes. *Nucleic Acids Research* **33**, D121-D124 (2005).
34. Lowe, T.M. & Eddy, S.R. tRNAscan-SE: A program for improved detection of transfer RNA genes in genomic sequence. *Nucleic Acids Research* **25**, 955-964 (1997).
35. Nawrocki, E.P. & Eddy, S.R. Infernal 1.1: 100-fold faster RNA homology searches. *Bioinformatics* **29**, 2933-2935 (2013).
36. She, R., Chu, J.S.C., Wang, K., Pei, J. & Chen, N.S. genBlastA: Enabling BLAST to identify homologous gene sequences. *Genome Research* **19**, 143-149 (2009).
37. Birney, E., Clamp, M. & Durbin, R. GeneWise and genomewise. *Genome Research* **14**, 988-995 (2004).
38. Wang, Y.P. *et al.* MCScanX: a toolkit for detection and evolutionary analysis of gene synteny and collinearity. *Nucleic Acids Research* **40**(2012).
39. Delcher, A.L., Phillippy, A., Carlton, J. & Salzberg, S.L. Fast algorithms for large-scale genome alignment and comparison. *Nucleic Acids Research* **30**, 2478-2483 (2002).
40. Ul Qamar, M.T., Zhu, X.T., Xing, F. & Chen, L.L. ppsPCP: a plant presence/absence variants scanner and pan-genome construction pipeline. *Bioinformatics* **35**, 4156-4158 (2019).
41. Li, H. Minimap2: pairwise alignment for nucleotide sequences. *Bioinformatics* **34**, 3094-3100 (2018).
42. Sedlazeck, F.J. *et al.* Accurate detection of complex structural variations using single-molecule sequencing. *Nature Methods* **15**, 461-+ (2018).

43. Chen, J. *et al.* Assessment of Glucosinolates in Chinese Kale by Near-Infrared Spectroscopy. *International Journal of Food Properties* **17**, 1668-1679 (2014).
44. Kumar, S., Stecher, G., Li, M., Knyaz, C. & Tamura, K. MEGA X: Molecular Evolutionary Genetics Analysis across Computing Platforms. *Molecular Biology and Evolution* **35**, 1547-1549 (2018).
45. Alexander, D.H., Novembre, J. & Lange, K. Fast model-based estimation of ancestry in unrelated individuals. *Genome Research* **19**, 1655-1664 (2009).
46. Price, A.L. *et al.* Principal components analysis corrects for stratification in genome-wide association studies. *Nature Genetics* **38**, 904-909 (2006).
47. Purcell, S. *et al.* PLINK: A tool set for whole-genome association and population-based linkage analyses. *American Journal of Human Genetics* **81**, 559-575 (2007).

Supplementary information

Supplementary Figures

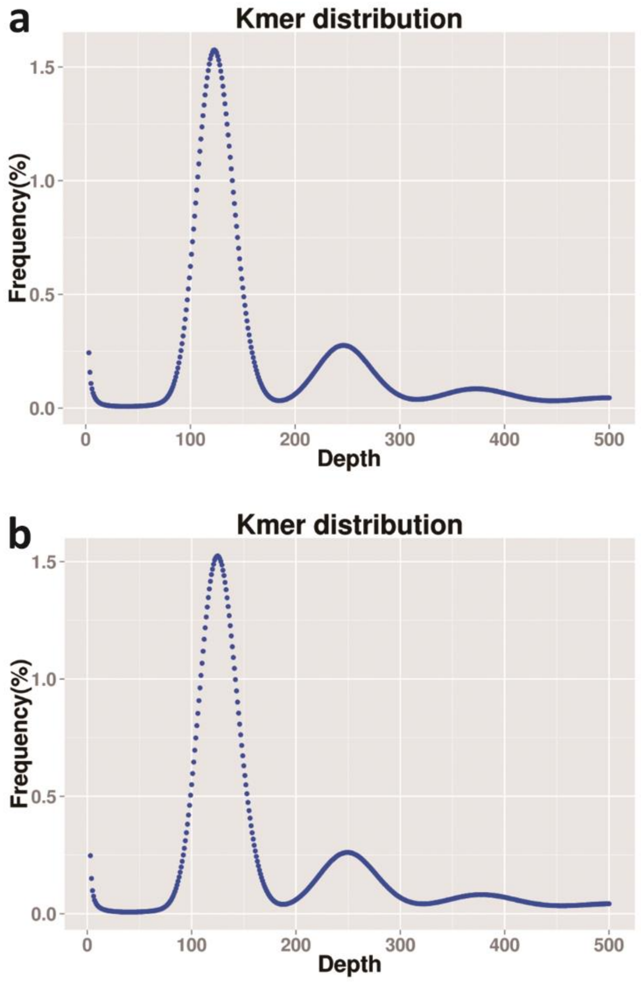

Supplementary Figure 1 Estimation of genome size, heterozygosity and repeated sequences percentage by using K-mer analysis of Illumina sequencing data. (**a**), T84-66; (**b**), AU213.

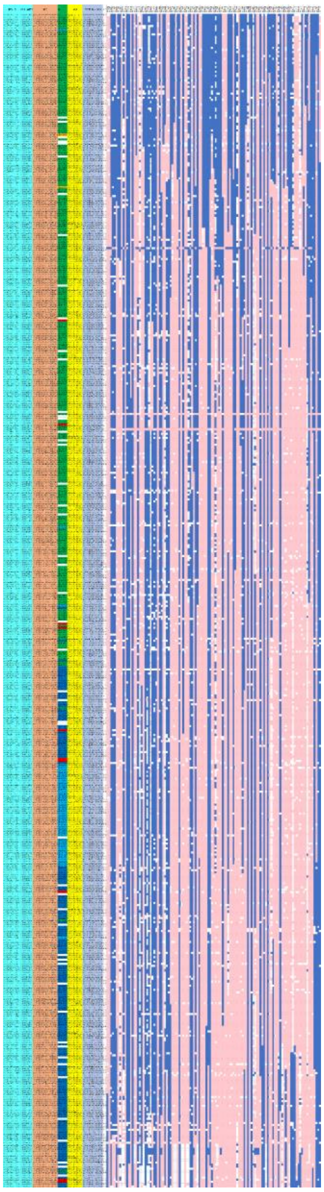

Supplementary Figure 2 Genome sequence organization of vegetable *B. juncea* (T84-66) with assessment using genome-ordered graphical genotypes and chromosome A01 as examples. Graphical genotypes are shown for transcriptome SNP markers scored across 134 lines of the VHDH mapping population with Heera alleles in coral, Veruna alleles in blue and missing scores in white. The sky blue, dark orange, multicolor, yellow and dark pick displayed gene locations in *B. juncea*, constructed colinear A01 subgenome, *A. thaliana*, *B. rapa* and York_AB position.

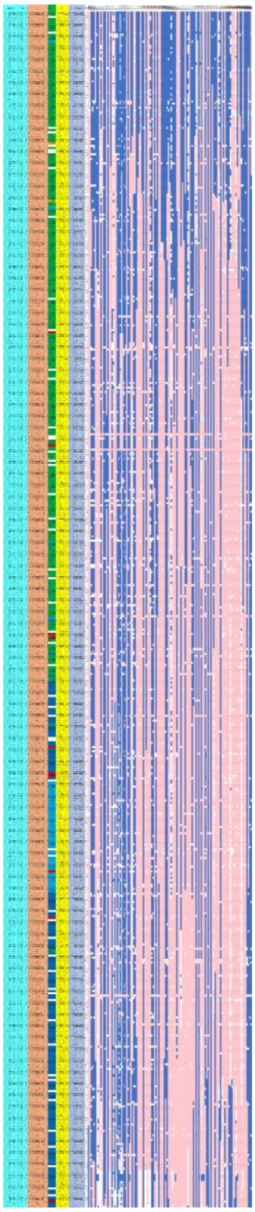

Supplementary Figure 3 Genome sequence organization of oilseed *B. juncea* (AU213) with assessment using genome-ordered graphical genotypes and chromosome A01 as examples. Graphical genotypes are shown for transcriptome SNP markers scored across 134 lines of the VHDH mapping population with Heera alleles in coral, Veruna alleles in blue and missing scores in white. The sky blue, dark orange, multicolor, yellow and dark pick displayed gene locations in *B. juncea*, constructed colinear A01 subgenome, *A. thaliana*, *B. rapa* and York_AB position.

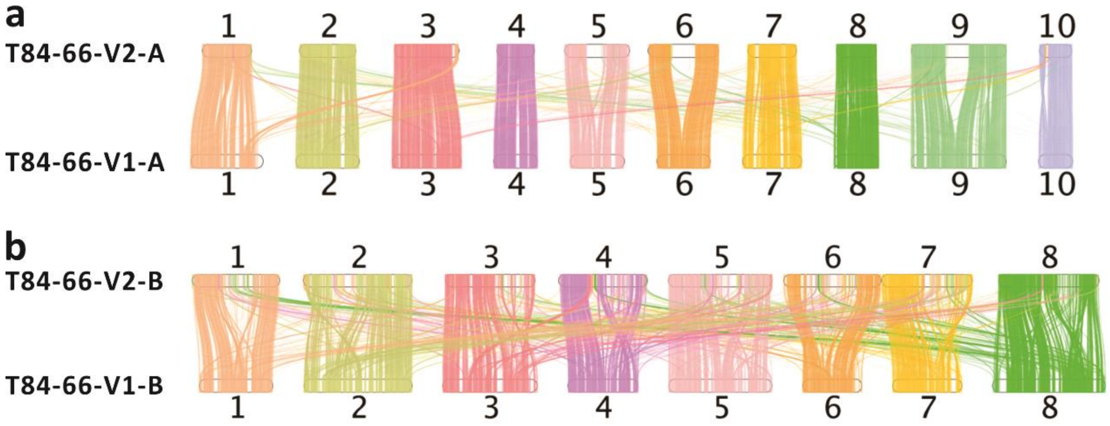

Supplementary Figure 4 Synteny analysis of between new assembly of vegetable (T84-66, V2) and published assembly of V1.0 (T84-66, V1). (**a**), A-subgenome; (**b**), B-subgenome.

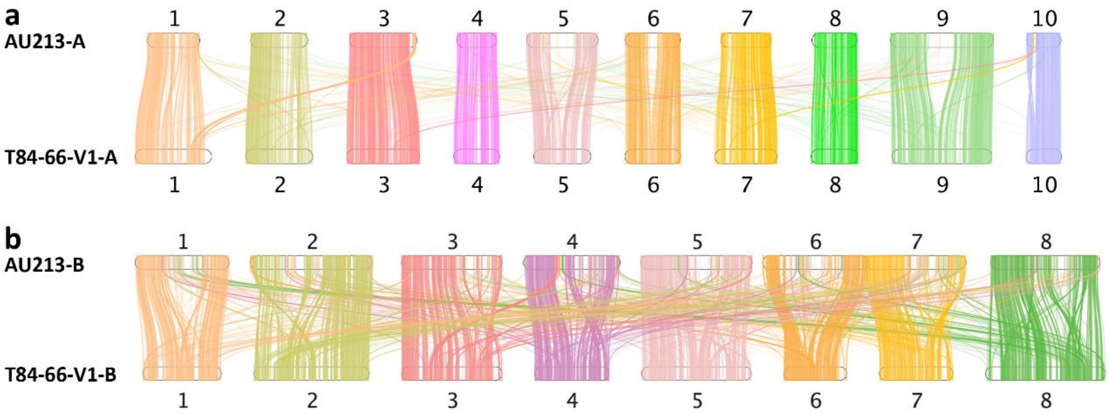

Supplementary Figure 5 Synteny analysis of between new assembly of vegetable (AU213) and published assembly of V1.0 (T84-66, V1). (**a**), A-subgenome; (**b**), B-subgenome.

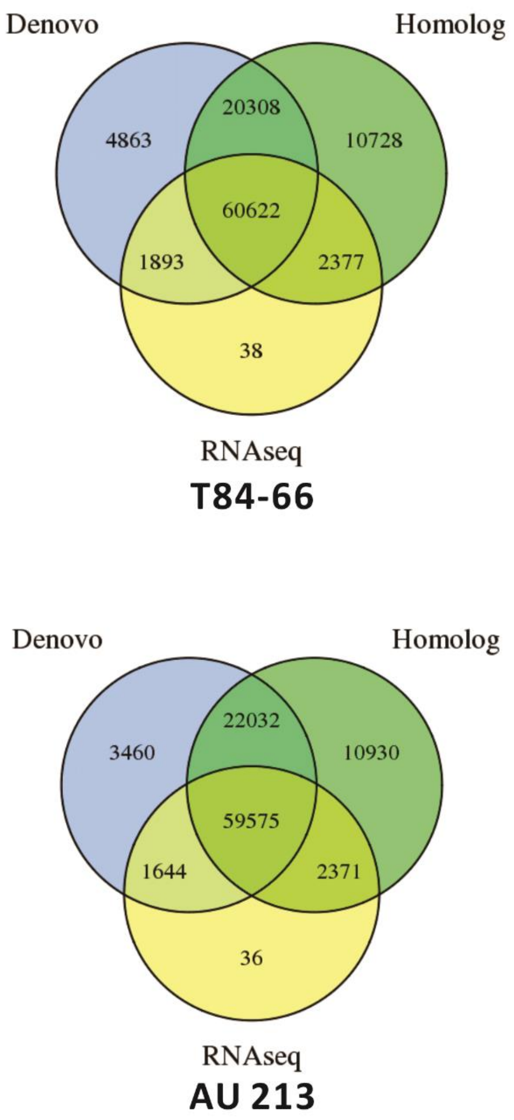

Supplementary Figure 6 Summary of gene annotation by RNA-seq and homological genes.

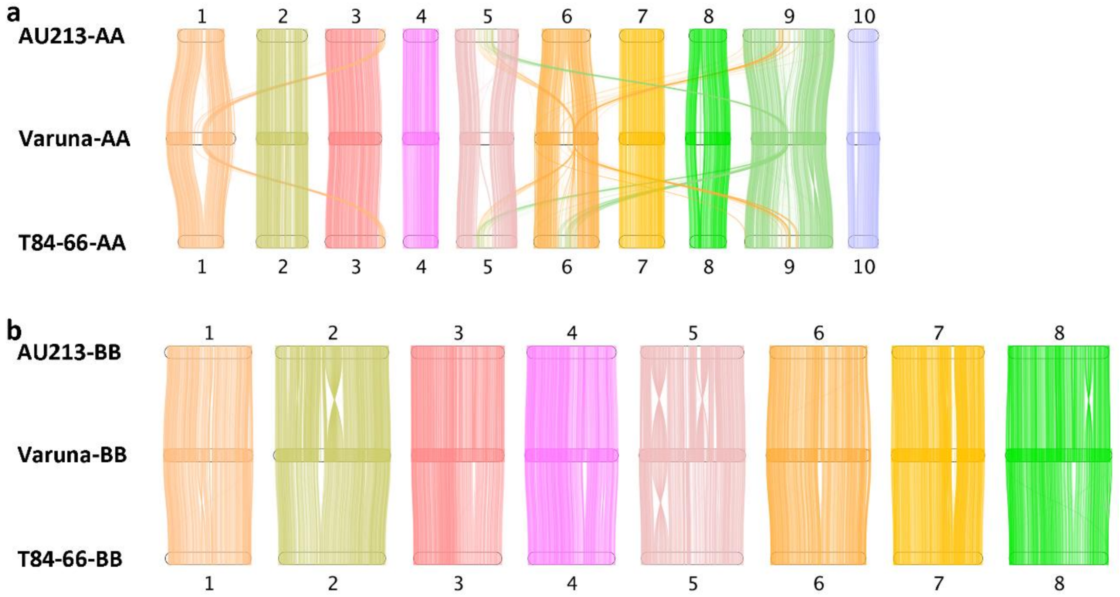

Supplementary Figure 7 Synteny analysis of between new assembly of A-subgenomes of vegetable (T84-66) and oilseed (AU213) *B. juncea* with a newly published oilseed *B. juncea* (2020).

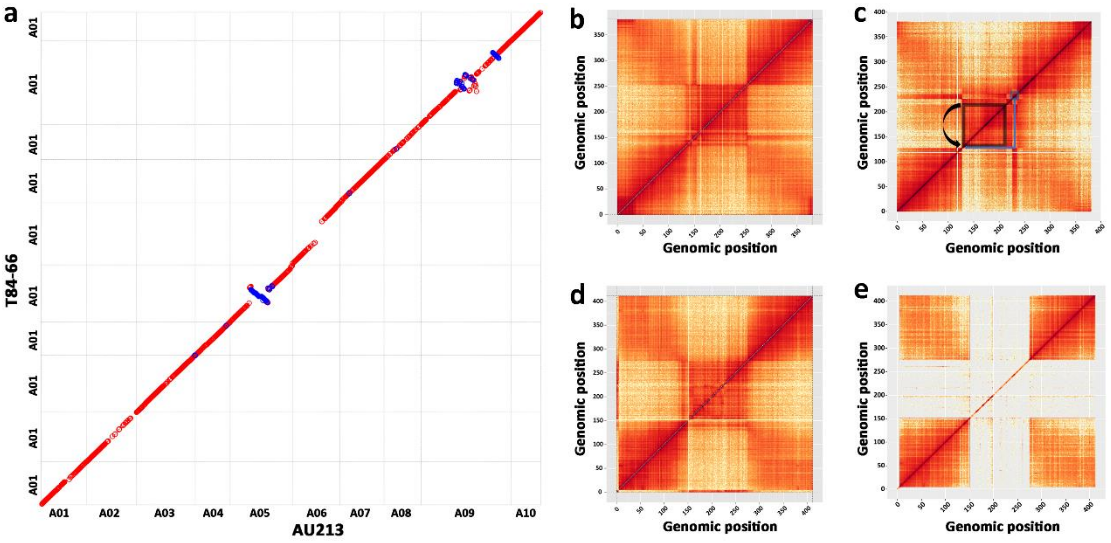

Supplementary Figure 8 Synteny analysis of between new assembly of A-subgenomes of vegetable (T84-66) and oilseed (AU213) *B. juncea*. (**a**) Synteny analysis of between new assembly of A sub-genomes of the T84-66 and AU213. (**b**, **c**) The identification of larger inversions in chromosome A05. The heatmaps show a chromatin interaction matrix demonstrating the mapping of T84-66 Hi-C data against the A05 chromosome of T84-66 (**b**), and against the A05 chromosome of AU213 (**c**). (**d**, **e**) The identification of larger deletions in chromosome A06. The heatmaps show a chromatin interaction matrix demonstrating the mapping of T84-66 Hi-C data against the A06 chromosome of T84-66 (**d**), and against the A06 chromosome of AU213 (**e**).

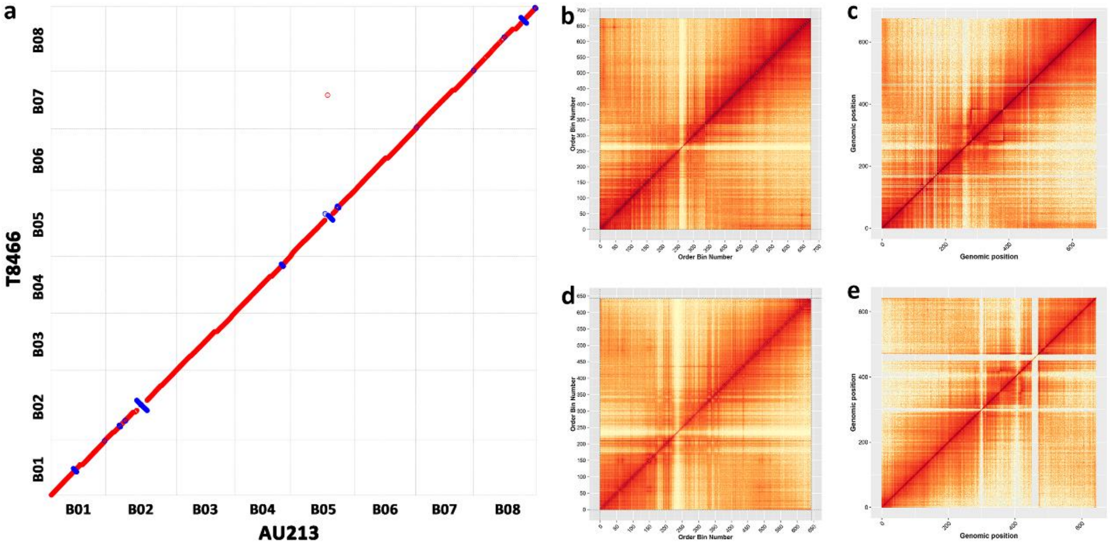

Supplementary Figure 9 Synteny analysis of between new assembly of B-subgenomes of vegetable (T84-66) and oilseed (AU213) *B. juncea*. (**a**) Synteny analysis of between new assembly of B sub-genomes of the T84-66 and AU213. (**b**, **c**) The identification of larger inversions in chromosome B02. The heatmaps show a chromatin interaction matrix demonstrating the mapping of T84-66 Hi-C data against the B02 chromosome of T84-66 (**b**), and against the B02 chromosome of AU213 (**c**). (**d**, **e**) The identification of larger deletions in chromosome B05. The heatmaps show a chromatin interaction matrix demonstrating the mapping of T84-66 Hi-C data against the B05 chromosome of T84-66 (**d**), and against the B05 chromosome of AU213 (**e**).

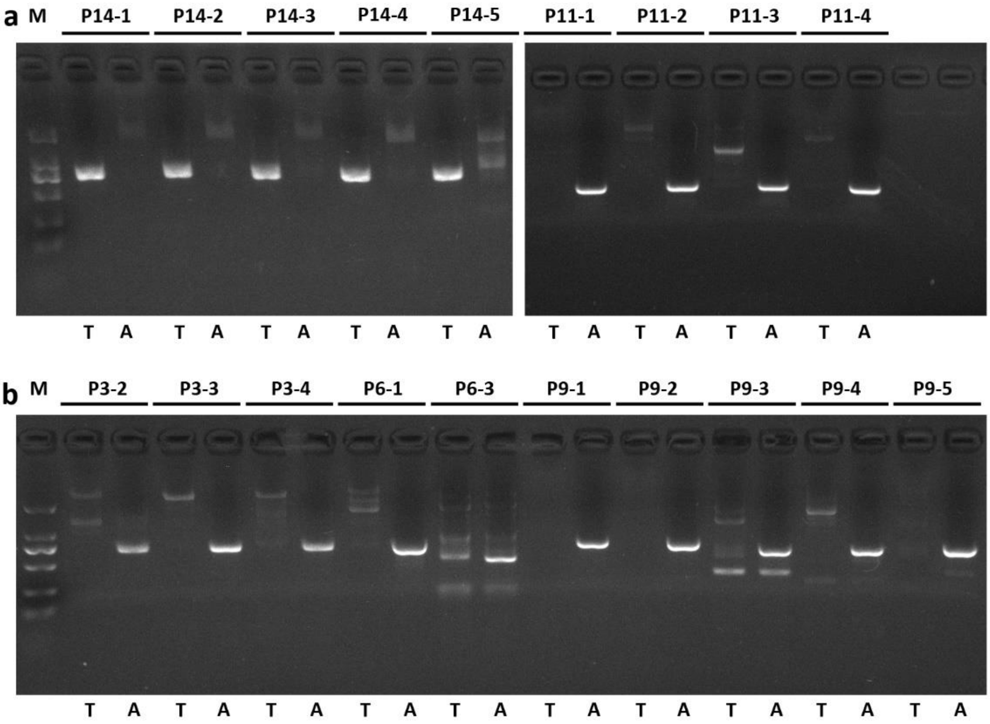

Supplementary Figure 10 Validation of selected PAVs in T84-66 and AU213 using PCR amplification. (**a**), PAVs in A-subgenome; (**b**), PAVs B-subgenome**.** T, T84-66; A, AU213.

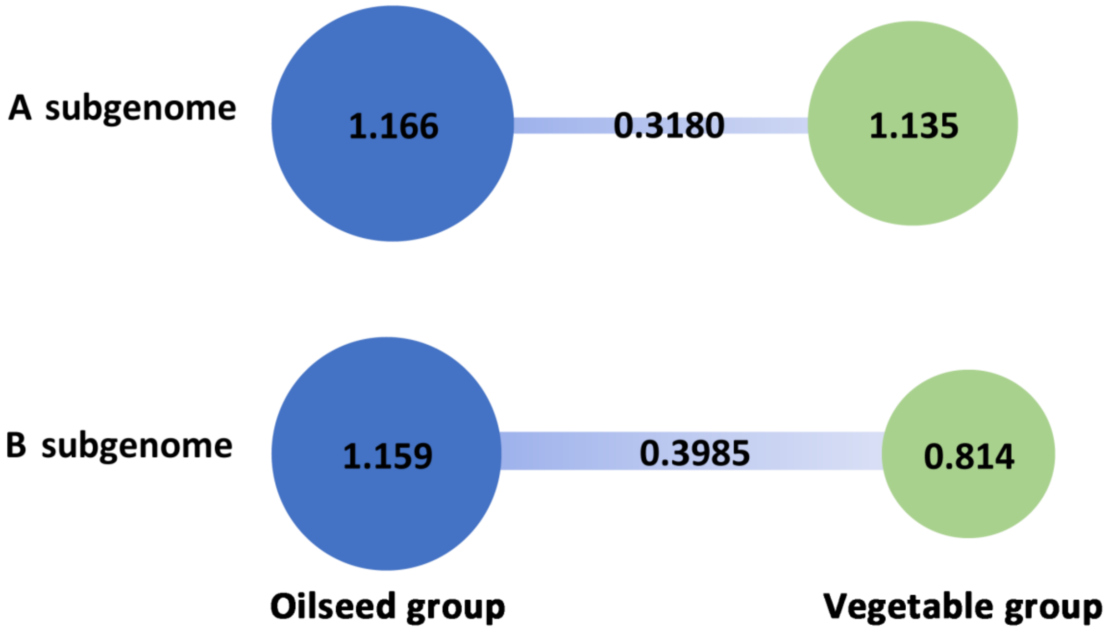

Supplementary Figure 11 *Fst* and π estimations of A and B subgenomes using resequencing population of *B. juncea*.

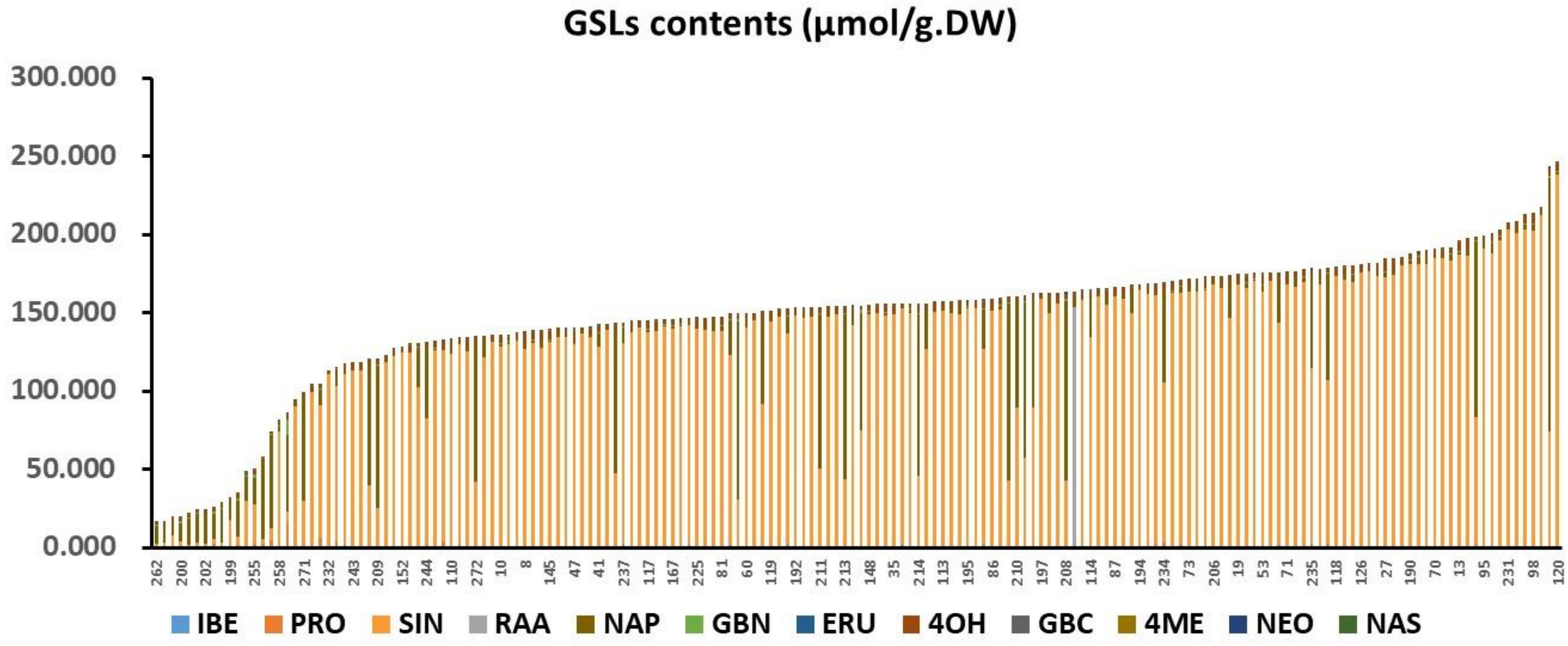

Supplementary Figure 12 Glucosinolates (GSLs) components analysis using HPLC in the resequencing population of *B. juncea*.

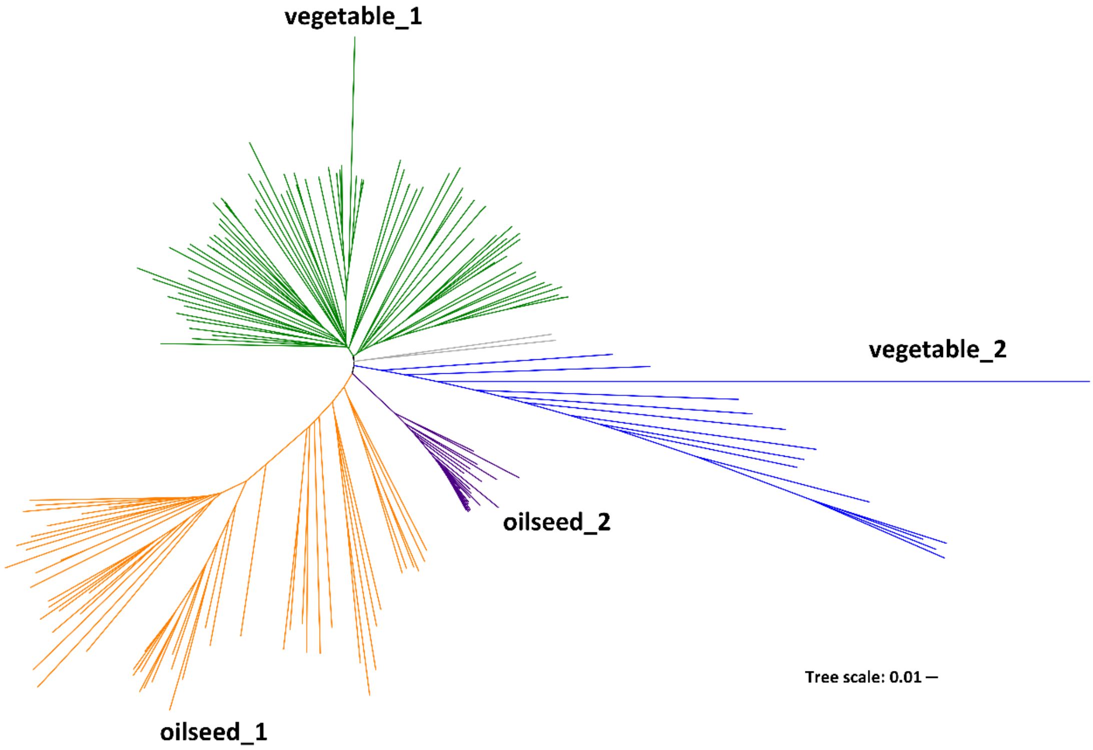

Supplementary Figure 13 A phylogenetic neighbor-joining tree constructed from 183 accessions of *B. juncea*.

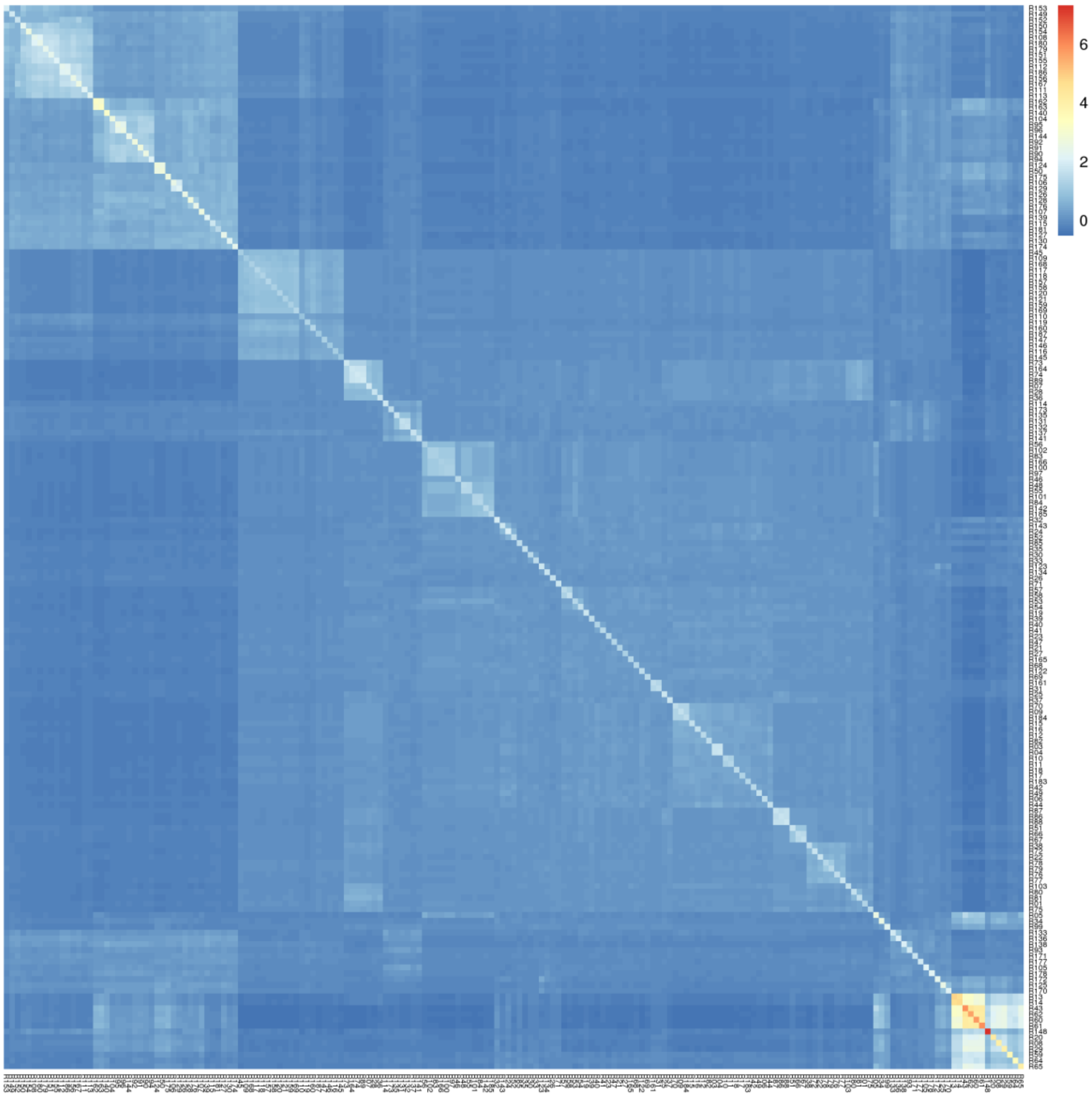

Supplementary Figure 14 Relative kinship analysis in the resequencing population of *B. juncea*.

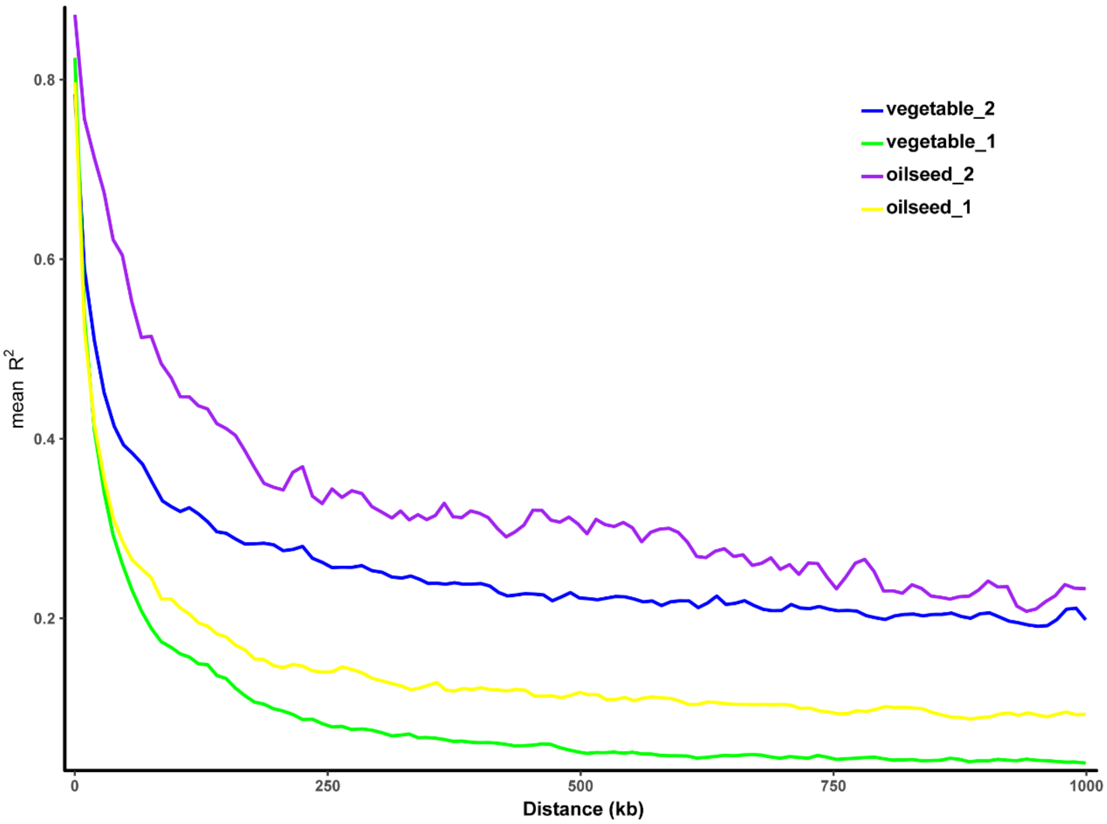

Supplementary Figure 15 LD decay distance in the resequencing population of *B. juncea*.

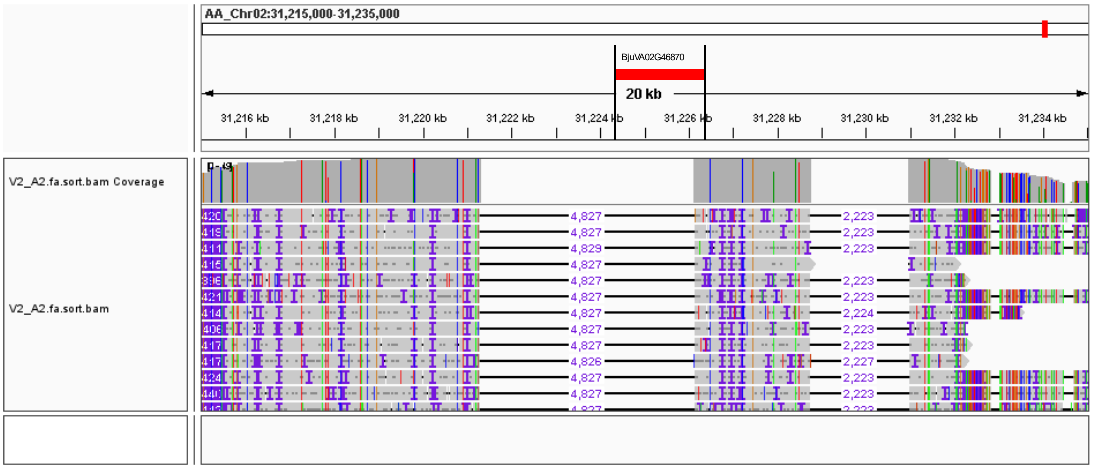

Supplementary Figure 16 A 4827-bp deletion covered the *MYB28* (*BjuVA02G46870.1*) in AU213.

Supplementary Tables

Supplementary Table 1. Summary of Illumina clean reads for two accessions of allopolyploid *B. juncea*

Variety	Library	Total reads (Gb)	Depth (X)	Q20 (%)	Q20 (%)
Vegetable (T84-66)	350 bp	146	156	97.54	93.21
Oilseed (AU213)	350 bp	147	152	97.85	94.08

Supplementary Table 2. Summary of PacBio clean subreads for two accessions of allopolyploid *B. juncea*

Variety	Reads Number	Total Reads Bases (bp)	Mean Reads Length (bp)	Max Reads Length (bp)
Vegetable (T84-66)	8,682,529	100,603,921,799	11,587	102,113
Oilseed (AU213)	9,551,240	105,043,643,250	10,998	90,330

Supplementary Table 3. Summary of subreads length distributions for two accessions of allopolyploid *B. juncea*

Variety	Length (bp)	Number	Total length (bp)	Average length (bp)
Vegetable (T84-66)	500-2000	1,201,956	1,496,198,839	1,245
	2000-4000	1,161,148	3,365,019,884	2,898
	4000-6000	850,621	4,224,215,170	4,966
	6000-8000	724,092	5,050,589,836	6,975
	8000-10000	634,681	5,698,633,898	8,979
	10000-12000	584,099	6,420,529,903	10,992
	12000-14000	570,652	7,413,890,009	12,992
	14000-16000	561,589	8,422,769,987	14,998
	16000-18000	497,747	8,442,831,439	16,962
	>18000	1,895,944	50,069,242,834	26,409
	Total	8,682,529	100,603,921,799	11,587
Oilseed (AU213)	500-2000	1,258,458	1,547,311,075	1,230
	2000-4000	1,225,860	3,585,739,541	2,925
	4000-6000	949,811	4,723,796,609	4,973
	6000-8000	826,982	5,770,182,432	6,977
	8000-10000	736,802	6,621,326,695	8,987
	10000-12000	760,851	8,385,532,268	11,021
	12000-14000	829,497	10,782,069,973	12,998
	14000-16000	721,423	10,790,651,287	14,957
	16000-18000	540,881	9,166,853,357	16,948
	>18000	1,700,675	43,670,180,013	25,678
	Total	9,551,240	105,043,643,250	10,998

Supplementary Table 4. Summary of Hi-C reads mapping

Variety	Library	Type	Number	Percentage (%)
Vegetable (T84-66)	N079	Unique paired alignments	177,580,975	100
		Valid interaction pairs	169,239,098	95.3
		Dangling end pairs	3,918,792	2.21
		Re-ligation pairs	1,111,743	0.63
		Self-cycle pairs	1,446,020	0.81
		Dumped pairs	1,865,322	1.05
	N103	Unique paired alignments	215,712,186	100
		Valid interaction pairs	203,425,799	94.3
		Dangling end pairs	4,991,513	2.31
		Re-ligation pairs	1,416,976	0.66
		Self-cycle pairs	3,588,134	1.66
		Dumped pairs	2,289,764	1.06
Oilseed (AU213)	N079	Unique paired alignments	78,731,155	100
		Valid interaction pairs	74,226,774	94.28
		Dangling end pairs	2,964,112	3.76
		Re-ligation pairs	541,178	0.69
		Self-cycle pairs	338,512	0.43
		Dumped pairs	660,579	0.84
	N103	Unique paired alignments	86,237,780	100
		Valid interaction pairs	81,197,749	94.16
		Dangling end pairs	3,151,608	3.65
		Re-ligation pairs	542,238	0.63
		Self-cycle pairs	596,728	0.69
		Dumped pairs	749,457	0.87
	N106	Unique paired alignments	9,546,919	100
		Valid interaction pairs	8,980,799	94.07
		Dangling end pairs	355,670	3.73
		Re-ligation pairs	61,292	0.64
		Self-cycle pairs	65,558	0.69
		Dumped pairs	83,600	0.88
	N110	Unique paired alignments	6,389,292	100
		Valid interaction pairs	6,021,286	94.24
		Dangling end pairs	229,360	3.59
		Re-ligation pairs	39,932	0.62
		Self-cycle pairs	44,144	0.69
		Dumped pairs	54,570	0.85

Supplementary Table 5. Summary of assembly by using Hi-C data

Vegetable (T84-66)			Oilseed (AU213)		
Group	Sequence number	Total sequence length	Group	Sequence number	Total sequence length
LG01	142	67,963,236	LG01	57	74,469,301
LG02	61	68,066,400	LG02	60	67,875,745
LG03	78	64,879,211	LG03	50	67,471,439
LG04	50	66,543,136	LG04	147	70,225,729
LG05	36	58,494,329	LG05	58	64,921,378
LG06	40	58,712,553	LG06	41	59,970,966
LG07	49	58,962,525	LG07	14	59,004,031
LG08	41	57,893,690	LG08	25	57,536,278
LG09	39	24,833,829	LG09	50	58,108,789
LG10	103	49,209,111	LG10	69	45,278,410
LG11	70	44,372,651	LG11	41	41,666,421
LG12	28	40,372,651	LG12	71	39,642,490
LG13	49	75,362,407	LG13	74	35,672,793
LG14	56	33,500,637	LG14	29	33,907,641
LG15	42	35,997,755	LG15	49	31,439,472
LG16	38	31,278,757	LG16	23	28,506,136
LG17	20	25,238,434	LG17	49	25,845,503
LG18	33	25,251,258	LG18	40	24,035,238
Total sequences clustered (ratio %)	975 (84.71)	866,707,337 (98.0)	Total sequences clustered (ratio %)	947 (89.68)	885,577,760(98.99)
Total sequences ordered and oriented (ratio %)	422 (43.28)	803,938,942 (90.67)	Total sequences ordered and oriented (ratio %)	351 (37.06)	812,650,330(91.76)

Supplementary Table 6. Summary of genome assemblies for *B. juncea*

	Vegetable (T84-66) V1.0-Illumina		Vegetable (T84-66) V1.0-Illumina+PacBio		Vegetable (T84-66) V1.0-Illumina+PacBio +BioNano		Vegetable (T84-66) V1.0-PacBio +Hi-C		Oilseed (Au213) V1.0-PacBio +Hi-C	
	Contig	Scaffold	Contig	Scaffold	Contig	Scaffold	Contig	Scaffold	Contig	Scaffold
Total length (bp)	640,594,512	701,290,321	760,709,244	784,227,516	955,000,958 (gap 194,291,714)		904,836,171		894,631,344	
Total number	48,985	11,891	32,581	10,784	10,581		1,093		1,056	
Max length (bp)	326,883	6,107,082	569,668	4,561,631	7,842,264		23,748,072		27,993,914	
N50 size (bp)	28,225	710,138	61,273	855,041	1,523,604		4,055,693		4,404,270	
N90 size (bp)	6,024	83,000	12,728	94,898	124,389		383,039		401,557	

Supplementary Table 7. BUSCO assessment of *B. juncea* genomes assembly

		Complete BUSCOs(C)	Complete and single-copy BUSCOs(S)	Complete and duplicated BUSCOs(D)	Fragmented BUSCOs(F)	Missing BUSCOs(M)	Total Lineage BUSCOs
Genome	AA-AU213	1397 (97.01%)	1216 (84.44%)	181 (12.57%)	12 (0.83%)	31 (2.15%)	1440
	AA-T84-66 (V2)	1391 (96.60%)	1214 (84.31%)	177 (12.29%)	10 (0.69%)	39 (2.71%)	1440
	AA-T84-66 (V1)	1271 (88.26%)	974 (67.64%)	297 (20.62%)	14 (0.97%)	155 (10.76%)	1440
	BB-AU213	1388 (96.39%)	1195 (82.99%)	193 (13.40%)	13 (0.90%)	39 (2.71%)	1440
	BB-T84-66 (V2)	1385 (96.18%)	1197 (83.12%)	188 (13.06%)	16 (1.11%)	39 (2.71%)	1440
	BB-T84-66 (V1)	1218 (84.58%)	972 (67.50%)	246 (17.08%)	24 (1.67%)	198 (13.75%)	1440
	AABB-AU213	1415 (98.26%)	257 (17.85%)	1158 (80.42%)	4(0.28%)	21 (1.46%)	1440
	AABB-T84-66 (V2)	1410 (97.92%)	283 (19.65%)	1127 (78.26%)	6 (0.42%)	24 (1.67%)	1440
	AABB-T84-66 (V1)	1408 (97.78%)	437 (30.35%)	971 (67.43%)	7 (0.49%)	25 (1.74%)	1440
Gene	AA-AU213	1370 (95.14%)	1183 (82.15%)	187 (12.99%)	16 (1.11%)	54 (3.75%)	1440
	AA-T84-66 (V2)	1391 (96.60%)	1193 (82.85%)	198 (13.75%)	12 (0.83%)	37 (2.57%)	1440
	AA-T84-66 (V1)	1145 (79.51%)	854 (59.31%)	291 (20.21%)	43 (2.99%)	252 (17.50%)	1440
	BB-AU213	1360 (94.44%)	1144 (79.44%)	216 (15.00%)	12 (0.83%)	68 (4.72%)	1440
	BB-T84-66 (V2)	1374 (95.42%)	1154 (80.14%)	220 (15.28%)	13 (0.90%)	53 (3.68%)	1440
	BB-T84-66 (V1)	1072 (74.44%)	844 (58.61%)	228 (15.83%)	43 (2.99%)	325 (22.57%)	1440
	AABB-AU213	1428 (99.17%)	73 (5.07%)	1355 (94.10%)	4 (0.28%)	8 (0.56%)	1440
	AABB-T84-66 (V2)	1428 (99.17%)	70 (4.86%)	1358 (94.31%)	4 (0.28%)	8 (0.56%)	1440
	AABB-T84-66 (V1)	1367 (94.93%)	341 (23.68%)	1026 (71.25%)	28 (1.94%)	45 (3.12%)	1440

Supplementary Table 8. Assembly assessment by CEGMA

Variety	Number of 458 CEGs* present in assembly	% of 458 CEGs present in assembly	Number of 248 highly conserved CEGs present	% of 248 highly conserved CEGs present
Vegetable (T84-66)	456	99.56%	247	99.60%
Oilseed (AU213)	457	99.78%	247	99.60%

Supplementary Table 9. Assembly assessment by Illumina-seq reads

Variety	Total reads	Mapped reads	Mapped (%)	Properly mapped reads	Properly mapped (%)
Vegetable (T84-66)	306,034,054	304,806,681	99.60	296,748,828	97.12
Oilseed (AU213)	305,900,639	304,586,132	99.57	293,874,876	96.18

Supplementary Table 10. Summary of repeated sequences annotation

Type	Vegetable (T84-66)			Oilseed (AU213)		
	Number	Length	percentage (%)	Number	Length	percentage (%)
ClassI	647810	422201021	46.66	634207	437398591	48.89
ClassI/DIRS	26829	30550646	3.38	34663	37663976	4.21
ClassI/LARD	240041	106424428	11.76	228997	122516633	13.69
ClassI/LINE	63238	24833086	2.74	60326	24838062	2.78
ClassI/LTR/Copia	110034	90650268	10.02	110712	90709256	10.14
ClassI/LTR/Gypsy	171676	189112959	20.9	167952	180879651	20.22
ClassI/LTR/Unknown	9107	4051136	0.45	9097	5356424	0.6
ClassI/PLE	9627	5363749	0.59	4564	3216209	0.36
ClassI/SINE	12504	3496460	0.39	10171	2542605	0.28
ClassI/TRIM	4302	5638652	0.62	7231	6380755	0.71
ClassI/Unknown	452	436977	0.05	494	437472	0.05
ClassII	122682	54052135	5.97	135840	59155991	6.61
ClassII/Crypton	25	1492	0	82	93623	0.01
ClassII/Helitron	11541	4860259	0.54	15389	6736986	0.75
ClassII/MITE	505	232104	0.03	288	191369	0.02
ClassII/Maverick	6532	4949913	0.55	8739	5718030	0.64
ClassII/TIR	92280	40346056	4.46	100264	42831067	4.79
ClassII/Unknown	11799	4289788	0.47	11078	4715399	0.53
PotentialHostGene	27621	8907550	0.98	33952	9817259	1.1
SSR	1366	443762	0.05	2314	966022	0.11
Unknown	54968	54339144	6.01	52497	35065779	3.92
Total	854447	511228113	56.5	858810	517404774	57.83

Supplementary Table 11. Summary of gene prediction

	Method	Program	Species	Gene number
Vegetable (T84-66)	*Ab initio*	Genscan	-	71,358
		Augustus	-	110,987
		GlimmerHMM	-	115,277
		GeneID	-	101,480
		SNAP	-	118,873
	Homology-based	GeMoMa	*Arabidopsis thaliana*	75,177
			Brassica napus	104,702
			Brassica oleracea	101,386
			Brassica rapa	89,006
	RNA-seq	PASA	-	54,097
		TransDecoder	-	73,088
		GeneMarkS-T	-	110,022
	Integrated	EVM	-	100,829
Oilseed (AU213)	*Ab initio*	Genscan	-	67,447
		Augustus	-	101,531
		GlimmerHMM	-	90,673
		GeneID	-	94,261
		SNAP	-	112,776
	Homology-based	GeMoMa	*Arabidopsis thaliana*	75,168
			Brassica napus	104,896
			Brassica oleracea	101,405
			Brassica rapa	89,328
	RNA-seq	PASA	-	43,855
		TransDecoder	-	74,415
		GeneMarkS-T	-	109,966
	Integrated	EVM	-	100,048

Supplementary Table 12. Characterization of genes in vegetable and oilseed of B. *juncea*

	Vegetable (T84-66)	Oilseed (AU213)
Gene number	100,829	100,048
Total gene length (bp)	225,784,251	219,640,205
Average gene length (bp)	2,239.28	2195.35
Total exon length (bp)	117,095,896	112,791,153
Average exon length (bp)	1,161.33	1,127.37
Total intron length (bp)	90,338,434	90,885,273
Average intron length (bp)	895.96	908.42

Supplementary Table 13. Summary of gene annotation by different databases

Dataset	Vegetable (T84-66)		Oilseed (AU213)	
	Gene number	Percentage (%)	Gene number	Percentage (%)
GO	74,286	73.68%	74,054	74.02%
KEGG	29,045	28.81%	30,921	30.91%
KOG	50,113	49.70%	49,556	49.53%
TrEMBL	98,793	97.98%	98,345,	98.30%
nr	98,922	98.11%	98,288	98.24%
All annotated	99,054	98.24%	98,453	98.41%

Supplementary Table 14. Summary of annotated genes of T84-66 and AU213 validated by multiple-blast and long-reads mapping

	Vegetable (T84-66)	Oilseed (AU213)
Total genes	100829	100048
No-hit with published version (2016)	33814	32730
Illumina transcriptome	6236	6251
ONT transcriptome	7104	6711
Blast with published genome (2020)	9472	10077
Orthologs with published genome (2020)	11859	12707
Assembly difference	13518	18973
Unmapped gene	8704	6040
Unmapped gene with annotation	8000	5472
Unmapped gene without annotation	704	568

Supplementary Table 17. Summary of noncoding RNA predication

Classification	Vegetable (T84-66)		Oilseed (AU213)	
	Number	Family	Number	Family
miRNA	310	24	349	24
rRNA	7,935	4	6,262	4
tRNA	2,511	23	2,344	24
snRNA	296	8	309	8
snoRNA	4,643	2	4,592	2

Supplementary Table 18. Summary of pseudo-gene predication

	Vegetable (T84-66)	Oilseed (AU213)
GeneWise	5,764	5,788
Total_Length	13,650,123	11,686,535
Average_Length	2,368	2,003

Supplementary Table 34. Statistics of variation density between subgenomes

Type	A-subgenome		B-subgenome	
	Amount	Density (Kb)	Amount	Density (Kb)
SNP	553,697	0.9620	511,918	1.6441
InDel	352,578	0.6126	336,664	1.0812
PAV	15,023	0.0261	9,744	0.0313
SV	7,249	0.0126	4,171	0.0134

Supplementary Table 36. Statistics of resequencing of 187 accessions of *B. juncea*

Sample ID	Total_Reads	Mapped (%)	Properly_mapped (%)	Ave_depth	Cov_ratio_1X (%)	Cov_ratio_5X (%)	Cov_ratio_10X (%)
R01	84530170	98.92	95.83	11	91.12	82.26	53.91
R02	77818644	98.93	95.77	10	90.75	81.8	49.66
R03	80497730	98.91	95.59	10	90.27	80.82	49.84
R04	99470530	98.74	95.54	13	90.69	84.89	68.09
R05	88101232	98.86	95.60	11	92.67	83.85	55.35
R06	92157182	98.78	95.39	12	90.43	83.19	60.29
R07	95598466	98.8	95.59	13	90.84	84.91	66.31
R08	86783144	98.35	94.36	11	90.08	79.44	54.19
R09	81055052	98.76	95.69	10	89.89	80.44	50.27
R10	89528568	98.78	95.68	11	90.01	81.94	57.54
R100	80653550	99.77	98.63	10	96.07	89.6	55.04
R101	76531370	99.26	97.13	10	92.77	84.31	49.52
R102	79213856	99.48	97.92	11	95.71	89.97	58.15
R103	81538794	98.77	95.46	11	89.79	81.69	54.77
R104	72047482	98.36	94.66	8	87.39	72.32	34.77
R105	79625892	98.49	95.10	10	88.08	78.05	47.32
R106	79356076	98.35	94.42	10	88.69	78.42	49.84
R107	88095432	98.51	95.10	11	88.67	80.45	54.7
R108	77915682	98.45	94.87	10	88.3	78.59	47.87
R109	78302970	98.76	95.62	9	89.68	77.57	43.04
R11	72863652	98.79	95.46	10	89.54	79.32	49.33
R110	70072956	98.72	95.64	8	92.26	74.99	34.41
R111	78135630	98.48	95.15	9	90.78	78.19	44.13
R112	84457208	98.44	95.23	11	89.05	81.21	54.21

R113	80456966	98.44	94.85	10	88.91	79.6	50.09
R114	84031118	98.66	95.74	11	89.73	82.55	55.28
R115	88851436	98.44	94.76	11	90.4	80.34	53.76
R116	78981938	98.69	95.35	10	90.03	79.49	48.16
R117	101717930	98.79	95.46	13	89.65	83.5	66.58
R118	97693128	98.82	95.55	12	89.49	82.35	61.21
R119	117163616	98.43	95.28	14	92	86.4	72.28
R12	114143658	98.96	95.70	14	90.87	85.32	71.65
R120	85472610	98.65	95.31	11	88.89	81.28	55.73
R121	94435614	98.76	95.56	11	90.15	82.19	57.28
R122	91300032	99	96.20	11	91.57	84.38	58.92
R123	92593744	98.85	95.61	12	89.59	82.28	59.5
R124	97509904	98.53	95.11	11	88.67	80.42	56.02
R125	84643856	98.52	94.87	10	88.55	78.49	49.36
R126	106651800	98.54	95.15	12	88.67	82.03	61.53
R127	102526758	98.55	95.15	12	88.91	81.62	59.53
R128	70170742	98.43	94.75	8	87.49	71.24	33.02
R129	75630378	98.48	94.94	9	88.65	75.74	41.42
R13	103524720	98.29	94.59	14	88.77	82.39	68.12
R130	95528172	98.54	95.13	11	88.66	80.52	56.45
R131	100451626	98.85	95.89	12	89.37	82.63	60.15
R132	77721070	98.87	95.95	9	88.78	76.86	40.77
R133	100154510	98.61	95.69	12	90.7	84.07	62.94
R134	93178122	98.95	96.23	10	89.13	80.54	50.38
R135	75622952	98.79	95.73	9	88.6	77.81	43.65
R136	90415352	98.57	95.31	11	89.27	81.14	55.03

R137	77612276	98.81	95.98	9	89.12	76.06	39.06
R138	86570532	98.63	95.32	10	88.43	77.51	45.62
R139	86639952	98.52	95.12	10	88.46	79.34	50.55
R14	88808634	98.43	94.45	12	88.43	80.73	58.5
R140	101521928	98.24	93.98	13	87.58	79.84	61.6
R141	88936286	98.89	96.17	10	90.56	81.63	49.88
R142	99895946	99.27	97.20	13	92.41	87.32	67.49
R143	107539586	98.95	96.35	13	89.53	83.9	65.65
R144	75283176	98.54	95.01	9	87.94	73.11	36.53
R145	97720528	98.67	95.28	12	90.86	83.79	63.35
R146	84747742	98.64	95.36	11	92.58	82.5	54.26
R147	78944870	98.65	95.11	10	89.25	79.56	50.34
R148	78520998	89.88	78.74	8	75.13	58.81	31.25
R149	93016872	98.42	94.85	11	88.44	80.46	56.57
R15	89119608	98.47	95.11	11	89.96	82.19	56.62
R150	90165752	98.47	94.93	11	88.39	79.76	54.03
R151	123923200	98.59	95.48	14	89.66	83.91	68.23
R152	85107820	98.57	95.28	10	89.95	79.71	49.34
R153	103052318	98.65	95.46	12	89.96	82.81	62.07
R154	95556056	98.36	94.84	12	88.62	81.11	58.58
R155	92573318	94.79	91.18	12	89.27	82.25	60.36
R156	75824908	95.52	92.17	9	88.85	76.61	40.86
R157	90058334	98.32	95.00	12	89.48	82.59	61.32
R158	105499624	97.04	93.87	13	89.96	83.76	66.48
R159	90342050	96.47	93.13	12	90.05	82.96	61.38
R16	90706162	98.48	95.38	12	90.59	83.62	59.58

R160	111383824	95.41	91.90	15	89.85	84.34	71.85
R161	93489518	98.01	95.10	11	93.85	85.28	59.98
R162	74507896	98.45	94.47	9	88.09	75.07	40.52
R163	93624852	98.43	94.56	11	88.79	80.31	56.8
R164	93005454	98.95	96.07	11	89.82	81.96	56.08
R166	75068988	99.58	98.29	11	96.1	90.96	60.3
R167	110942876	98.17	94.94	14	89.24	83.78	70.73
R168	76168680	98.77	96.15	9	89.43	75.49	38.31
R169	110451914	98.86	96.14	13	89.89	83.45	65.26
R17	77039794	98.71	95.58	10	91.32	81.69	50.57
R170	81572208	97.72	94.37	9	88.4	74.55	42.68
R171	113137752	98.57	95.60	13	90.21	83.62	66.55
R172	92237042	98.55	95.52	11	89.13	80.2	52.87
R173	119728430	98.87	96.56	14	90.44	85.19	68.89
R174	101050734	98.45	95.70	12	88.9	81.25	58.38
R175	108534562	98.38	95.48	12	90.14	81.67	59.81
R176	87516148	98.41	95.19	10	87.6	77.62	48.68
R177	81933732	98.5	95.52	10	87.87	76.66	44.75
R178	94832240	98.43	95.45	11	88.25	80.46	55.59
R179	81087698	98.25	95.22	10	88.84	78.14	47.18
R18	76968564	98.72	95.80	10	90.87	80.51	46.75
R180	75814408	96.86	93.74	9	87.93	76.36	42.62
R181	75750690	96.93	93.47	10	90.29	77.75	45.55
R182	77788620	97.78	93.11	19	40.23	36.81	33.4
R184	76818702	98.75	96.33	8	89.46	74.49	33.69
R185	90776360	99.11	97.33	12	94.05	88.7	63.08

R186	77389702	98.37	95.32	9	88.47	75.67	39.74
R187	76285448	98.6	95.91	9	88.84	76.75	40.88
R19	94787300	98.56	95.49	12	90.72	83.98	63.67
R20	84770880	98.29	94.50	11	87.24	78.74	54.04
R21	71937172	98.97	96.35	9	89.34	75.83	37.77
R22	87365508	98.74	95.59	11	89.12	81.43	56.08
R23	83223180	99.03	96.23	11	91.04	83.16	54.76
R24	93255824	98.92	96.38	11	92.08	85.78	60.52
R25	76375636	98.17	95.21	10	89.28	78.79	47.12
R26	78086040	98.84	95.87	9	89.74	78.08	43.07
R27	81885642	98.96	95.90	10	91.74	81.3	48.81
R28	73425382	98.88	95.63	9	89.48	78.31	43.52
R29	73688920	98.39	94.60	9	89.15	74.7	40.6
R30	74637274	98.9	96.09	9	92.45	79.52	41.39
R31	91102616	98.82	95.80	11	93.6	84.27	57.6
R32	103531418	98.83	95.85	13	91.28	86.03	69.13
R33	84841924	98.7	95.78	11	91.97	84.95	57.93
R34	79607446	98.51	94.68	11	89.08	79.59	52.17
R35	86821498	98.99	96.38	10	91.45	81.47	47.3
R36	76346736	98.74	95.46	10	89.61	79.93	48.83
R37	74488124	98.9	96.34	9	92.01	80.82	44.76
R38	88803882	98.78	95.72	11	90.65	83.62	58.27
R39	91649916	98.86	96.09	11	91.12	84.19	59.06
R40	81782014	98.38	95.20	10	90.07	80.62	50.41
R41	77140698	98.85	95.81	10	89.76	79.16	45.55
R42	78743984	98.86	95.96	9	91.98	80.14	45.32

R43	92164268	98.2	94.01	12	86.82	78.89	58.75
R44	75780688	99.01	96.20	9	90.12	78.21	41.41
R45	70685700	98.86	95.58	8	88.73	74.64	36.09
R46	80263668	98.47	95.89	11	91.24	84.64	57.75
R47	86495456	98.91	96.14	11	90.82	82.96	55.04
R48	84510484	99.49	97.54	11	93.41	87.37	59.21
R49	87439856	98.98	96.12	11	91.04	83.92	58.86
R50	96390840	98.54	95.00	11	88.4	79.8	54.52
R51	79649224	98.84	95.58	10	90	80.79	49.12
R52	88294842	98.99	95.89	11	91.03	84.1	59.61
R53	93425138	99.11	96.30	12	91.6	84.08	61.1
R54	82933934	99.11	96.12	10	90.24	81.93	51.66
R55	75278996	99.03	96.75	11	93.34	86.32	55.1
R56	84089502	99.37	97.43	10	94.19	84.78	49.23
R57	87062800	99.03	96.42	11	91.18	83.55	54.46
R58	112281058	99.02	96.52	14	91.77	87.5	72.07
R59	79151140	98.48	95.36	9	87.41	75.98	42.54
R60	83348028	98.34	94.46	10	86.1	76.01	48.09
R61	80605376	98.02	94.40	10	86.83	75.58	46.41
R62	85190534	98.14	94.04	11	87.38	77.26	51.74
R63	74156582	97.44	92.42	16	42.44	35.9	31.13
R64	82530984	98.66	95.22	10	87.8	78.19	48.09
R65	85700404	98.47	94.57	12	87.97	80.74	57.97
R66	81465524	98.82	95.91	10	90.49	80.71	48.53
R67	89106674	98.72	95.74	11	90.52	83.43	57.96
R68	82393620	99.06	96.24	11	91.89	84.06	55.2

R69	75290136	98.89	96.14	9	90.01	78.23	41.76
R70	69530960	98.75	95.35	9	89.41	76.35	38.5
R71	85662056	98.94	96.00	10	90.49	79.73	48.7
R72	98437980	98.91	96.23	13	92.3	87.42	69.28
R74	87593590	98.89	95.82	10	89.54	80.63	51.89
R75	96784384	98.7	95.81	13	90.98	85.28	68.04
R76	94453794	98.65	95.76	13	90.18	84.7	66.65
R77	86903968	98.65	95.62	11	89.31	81.47	54.91
R78	81059050	98.85	96.09	10	90.17	79.85	46.55
R79	91164246	98.8	95.73	12	89.77	83	62.18
R80	83274674	98.74	95.76	11	88.26	80.65	55.98
R81	86053978	98.73	95.87	11	89.97	82.96	58.76
R82	82074560	98.86	95.94	10	89.72	80.24	47.89
R83	127847230	99.65	98.22	18	95.99	94.04	88.34
R84	92681920	99.05	96.97	12	93.01	87.52	65.54
R85	79914914	98.82	95.64	10	91.21	80.73	50.05
R86	86546880	98.96	96.46	11	90.92	83.27	53.86
R87	80744610	98.65	96.29	10	90.86	82.19	49.83
R88	98322688	98.82	96.37	12	91.07	84.23	62.38
R89	87122424	98.87	96.06	11	89.68	81.99	56.19
R90	80412992	98.43	94.83	9	88.41	76.41	43.55
R91	77311384	98.39	94.59	9	87.34	75.64	43.11
R92	98195048	98.39	94.85	12	88.37	80.99	60.25
R93	97189772	98.61	95.24	12	89.69	82.4	58.82
R94	98341000	98.42	94.62	12	89.7	80.9	58.08
R95	77895714	97.56	93.26	9	89.38	76.12	43.44

R96	85179772	97.66	93.33	10	89.62	78.05	49.21
R97	92723984	99.59	98.24	12	96.33	92.63	70.06
R98	80821622	89.58	78.32	13	45.75	37.11	29.06
R99	88588622	98.8	95.84	11	90.99	81.05	53.47
R73	88941584	98.72	95.84	11	89.4	81.04	55.61
R165	96857776	97.95	95.39	13	91.28	83.6	64.79
R183	98930968	98.82	96.38	13	88.89	80.36	61.23
